# Ancient allopatry and ecological divergence act together to promote plant diversity in mountainous regions: evidence from comparative phylogeography of two genera in the Sino-Himalayan region

**DOI:** 10.1186/s12870-023-04593-1

**Published:** 2023-11-17

**Authors:** Junchu Peng, Xiangguang Ma, Hang Sun

**Affiliations:** 1 CAS Key Laboratory for Plant Diversity and Biogeography of East Asia, Kunming Institute of Botany, Chinese Academy of Sciences, 650201 Kunming, China; 2 University of Chinese Academy of Sciences, 100049 Beijing, China

**Keywords:** Comparative phylogeography, *Beesia*, *Megacodon*, Sino-Himalayan, Ecological divergence, Habitat fragmentation

## Abstract

**Background:**

How geographical isolation and ecological divergence act together to promote plant diversity in mountainous regions remains largely unknown. In this study, we chose two genera comprising a small number of species distributed in the Sino-Himalayan region, *Megacodon* (Gentianaceae) and *Beesia* (Ranunculaceae), which both exhibit a fragmented distribution pattern and are found across a wide range of elevations. By summarizing their common patterns of speciation and/or divergence processes, we aim to understand how environmental changes accelerated lineage diversification in the Sino-Himalayan region through ancient allopatry and ecological divergence.

**Results:**

Using ddRAD-seq, chloroplast genome sequences, and specific molecular markers, we studied the phylogenetic relationships, population structure, and historical biogeography of *Beesia* and *Megacodon*. Both genera began to diverge from the late Miocene onwards, with ancient allopatry at lower elevations formed narrow-range species or relict populations. Mantel tests between genetic distance and climatic, elevational, or geographic distance revealed an isolation-by-distance pattern in *Beesia* and *Megacodon stylophorus*. *Megacodon* showed two clades occupying entirely different altitudinal ranges, whereas *Beesia calthifolia* exhibited a genetic divergence pattern along an elevation gradient. Furthermore, we conducted morphological measurements on *Beesia calthifolia* and found that different elevational groups had distinct leaf shapes.

**Conclusions:**

The regional disjunctions of plant groups in the Sino-Himalayan region are drastic and closely related to several biogeographic boundaries. As a consequence of major geological and climate change, ecological divergence when different elevations are colonized often happens simultaneously within plant groups. Although habitat fragmentation and parapatric ecological divergence each spur speciation to different extents, a combined effect of these two factors is a common phenomenon in the Sino-Himalayan region.

**Supplementary Information:**

The online version contains supplementary material available at 10.1186/s12870-023-04593-1.

## Background

A plant species usually occupies a specific habitat type because of niche conservatism [1, 2] and tracks suitable ecological niches into a new geographical area (i.e., disperses). Therefore, niche conservatism promotes habitat fragmentation by limiting adaptation to new environments differing from its ancestral niche [3]. However, plant species can also colonize new ecological niches through adaptive changes, resulting in ecological divergence [4]. Mountains provide diverse habitat types and high habitat heterogeneity in topographically complex regions. The patterns of divergence of a plant species in mountainous regions are, therefore, closely related to habitat fragmentation in its ancestral distribution range and its ability to adapt to a novel ecological niche [5, 6]. Habitat reduction or fragmentation in mountainous regions is directly associated with geological and climatic events under natural circumstances [7, 8]. These events may have created a geographical or climatic barrier and can cause local extinctions, but relict populations may be isolated in relatively stable environments (i.e., refugia), resulting in an interruption of gene flow and giving rise to allopatric speciation. Ecological divergence is closely related to environmental gradients which mountain regions can provide, and it can also be promoted by geological and climatic changes. Although both habitat fragmentation and parapatric ecological divergence have been proposed to spur speciation, disentangling the relative effects of those processes in mountain regions is still challenging [9, 10].

The Sino-Himalayan region is a temperate biodiversity hotspot with a high level of species endemism [11–13]. It is also a modern distribution and diversification center for temperate flora in the northern hemisphere. The high level of vascular plant richness and endemism in this region have been enhanced by tectonic movement and climatic oscillation since the Miocene [12, 14–16]. Allopatric isolation has been proven as an important mechanism for promoting speciation or intra-specific divergence of many plant groups in this region (e.g., [17–19]). Several important biogeographic lines have been proposed in this region [20–22]. In addition to highly fragmented habitats, this region is also characterized by alpine valleys (2000–6000 m a.s.l.) with highly heterogeneous environments per a very small geographical range. These can promote ecological divergence and local adaptation of different populations of a species. However, few studies have focused on the role of the ecological shift in this region’s speciation or intra-specific divergence [23, 24]. In addition, the same elevation range in different mountain chains in the Sino-Himalayan region may have completely different environments. For example, an area at an elevation of 2000 m in the Sino-Himalayan region can be a dry hot valley, but it can also be a very humid forest. In the heterogenization process of the environment in this region, plants might have to migrate vertically to find suitable habitats to avoid local extinction, giving rise to allopatric divergence and ecological differentiation simultaneously between populations in stable (or ancestral) environments and populations in a variable environment. But until now, this hypothesis has not been fully tested in this region, although some studies have found a simultaneous allopatric and ecological divergence between sister species (or lineages) (e.g., [25–27]).

**Fig. 1:**
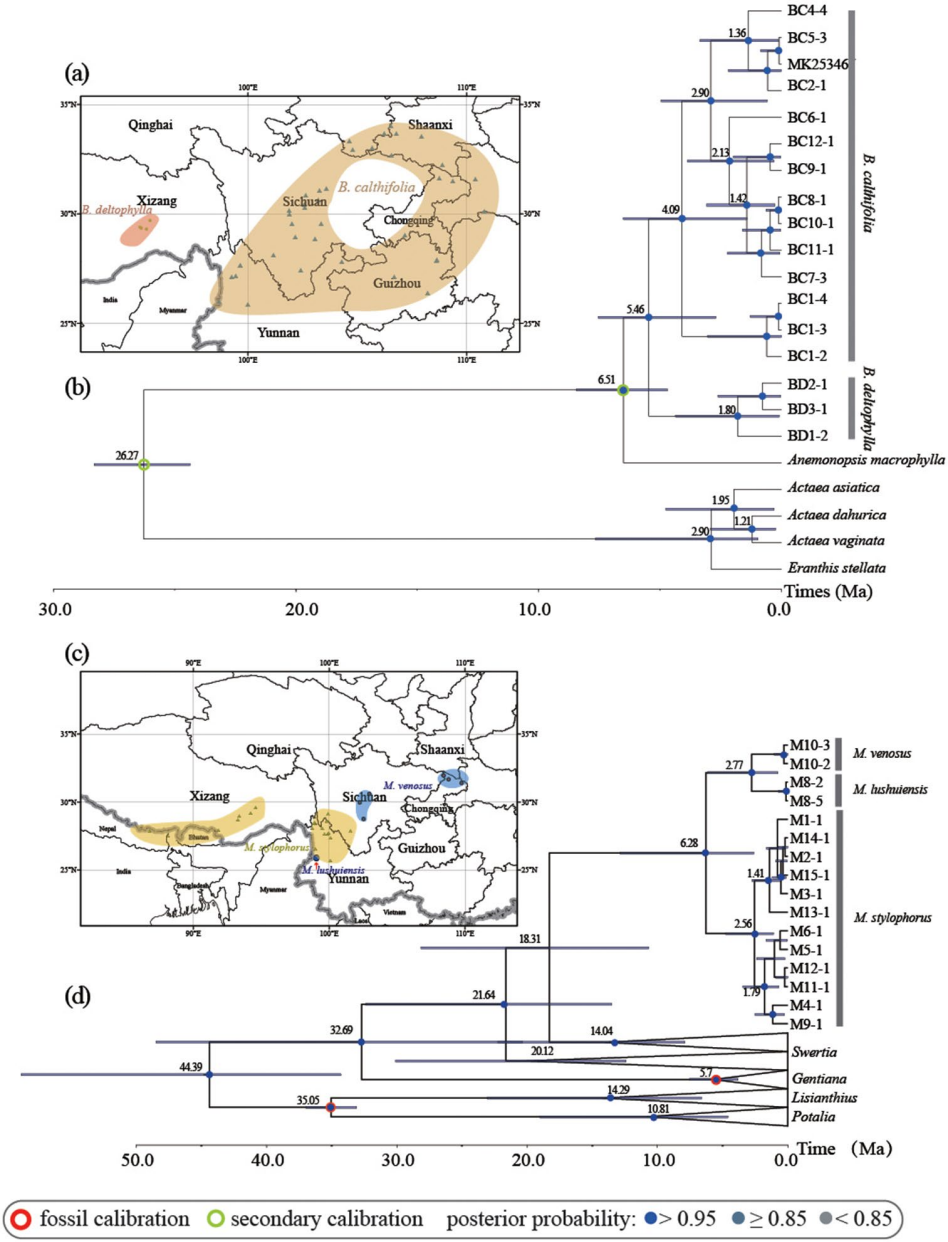
Geographic distribution and phylogenetic chronogram. (**a**) and (**c**), geographic distribution based on records of specimens and *Flora of China* for *Beesia* and *Megacodon* respectively. Shaded areas indicate the distribution areas. Phylogenetic chronogram of *Beesia* (**b**) and *Megacodon* (**d**) produced by BEAST. The *Beesia* data were based on 78 chloroplast CDSs, while the *Megacodon* data were based on ITS, *atpB-rbcL* and *trnL-trnF* sequences. Red circle indicates the fossil calibration, green circle indicates the secondary calibration. Blue dots are on well-supported nodes (posterior probabilities > 0.95), grey blue dots are on moderately supported (posterior probabilities ≥ 0.85), and grey dots are on weakly supported (posterior probabilities < 0.85)

Endemic genera or an endemic clade within genera in a mountain region may be an ideal system for studying specific speciation or divergence processes. Phylogeographic studies of endemic species or genera could reveal their distribution ranges and ecological niche evolution over evolutionary timescales, thereby helping us to understand and explore the relationship between speciation and the environmental changes that took place in a given geographical area [28]. Here, we present population assessments of the evolutionary history of two genera that are mainly distributed in the Sino-Himalayan region: *Megacodon* (Hemsl.) H. Smith and *Beesia* Balf. f. & W.W. Sm. These two genera are representative temperate groups that favor a humid environment in or near temperate forests. *Megacodon* belongs to the tribe Swertiinae of Gentianaceae and contains three species, *M*. *stylophorus*, *M. lushuiensis*, and *M*. *venosus* [29–31], while *Beesia* belongs to tribe Cimicifugeae in Ranunculaceae and has two species: *B*. *calthifolia* and *B*. *deltophylla*. [32–37]. These two genera show similarities in their whole biogeographic distribution ranges (Fig. 1a and c), and both offer a fragmented distribution pattern. These two genera are distributed in regions with relatively stable forest ecosystems, such as central China and mountain regions around Sichuan Basin which harbor many relict genera or species. They also both occur in the Hengduan Mountains region, which has experienced drastic tectonic movement and climatic oscillation. Another similarity is that these two genera both occur at a wide range of elevations and grow in (or near) the understory of different temperate forest types.

The sister species (or clade) pairs in *Megacodon* occupy very different elevation ranges, grow within completely different vegetation types, and show significant differentiation in their microhabitat. *Megacodon stylophorus* usually grows at an elevation of above 3600 m and can occur in a variety of alpine habitats, but the two lowland species (*M. lushuiensis* and *M*. *venosus*) usually occur at elevations of below 2000 m, which are greatly affected by human activities. In contrast, the two *Beesia* species have no obvious differentiation in elevation ranges and microhabitat (both grow in the understory of moist forests). However, *B. calthifolia* occupies a very large elevation range (from 1700 to 3900 m) and can grow in the understory of different forest types. Therefore, ecological divergence may occur within the species *B. calthifolia*. Although different plant groups have their own characteristics, different plant groups may share a similar divergence pattern because they share the same topographic and climatic histories in the Sino-Himalayan region.

In this study, we tried to uncover the biogeographic histories of *Megacodon* and *Beesia* using various data (ddRAD-seq, chloroplast genome, molecular fragment, and morphological data) to identify similarities in the evolutionary histories of the two genera. The ecological divergence in *Megacodon* is evident according to our previous study [30]. In order to compare the ecological divergence of these two genera, we perform a study about whether the divergence process in *B. calthifolia* may have been affected by niche divergence. With this study, we want to test the hypothesis that both habitat fragmentation and ecological adaptation widely influence speciation or divergence processes in the Sino-Himalayan region and their divergence processes are closely related to environmental heterogeneity in this region.

## Results

### Phylogenetic relationships and molecular dating

The length of whole chloroplast genome of *Beesia* were ranged from 156,961 to 158,274 (details in Additional file 2: Table S11). The alignment of the 78 non-duplicated chloroplast coding genes for *Beesia* was 69,256 bp in length. According to plastid phylogenomics, *Beesia deltophylla* diverged from *B*. *calthifolia* in the late Miocene to early Pliocene [5.46 Ma; 95% highest posterior density (HPD): 2.69–7.54 Ma Fig. 1b]. The BC1 population from the western edge of the Sichuan Basin represents the earliest evolutionary branching in *B*. *calthifolia* (Fig. 1b). Further genetic divergences of *B*. *calthifolia* took place in the late Pliocene to early Pleistocene (Fig. 1b).

*Megacodon* may have originated during the early Miocene (stem age: 18.9 Ma, HPD: 10.67–28.14 Ma) and diverged into two clades which occurred at different elevations during the Middle to Late Miocene (crown age: 6.28 Ma, HPD: 2.60–12.87 Ma; Fig. 1d). *Megacodon stylophorus* further diverged into two geographical lineages at 2.56 Ma (1.09–4.77 Ma), and *M*. *venosus* and *M*. *lushuiensis* also diverged at a similar time (2.77 Ma, HPD: 0.75–6.17 Ma).

### Phylogeographical structure

For 119 individuals of *Beesia* (82 individuals of 12 populations of *Beesia calthifolia*, 37 individuals of *B. deltophylla*), a total of 1,279,331,182 clean reads were generated after filtering, with a sequencing depth of 6.02 to 15.49 X (Additional file 2: Table S4). A total of 666 SNPs were obtained for further analysis of *Beesia* (dataset B119). For all the individuals of the three *Megacodon* species, a total of 881,394,581 clean reads were generated after filtering, with a sequencing depth of 6.73 to 22.14 X (Additional file 2: Table S3). After SNP discovery using Stacks, 278 SNPs were obtained for all the three *Megacodon* species (dataset M155). 1777 SNPs were obtained for *M*. *stylophorus* (dataset M131).

**Fig. 2:**
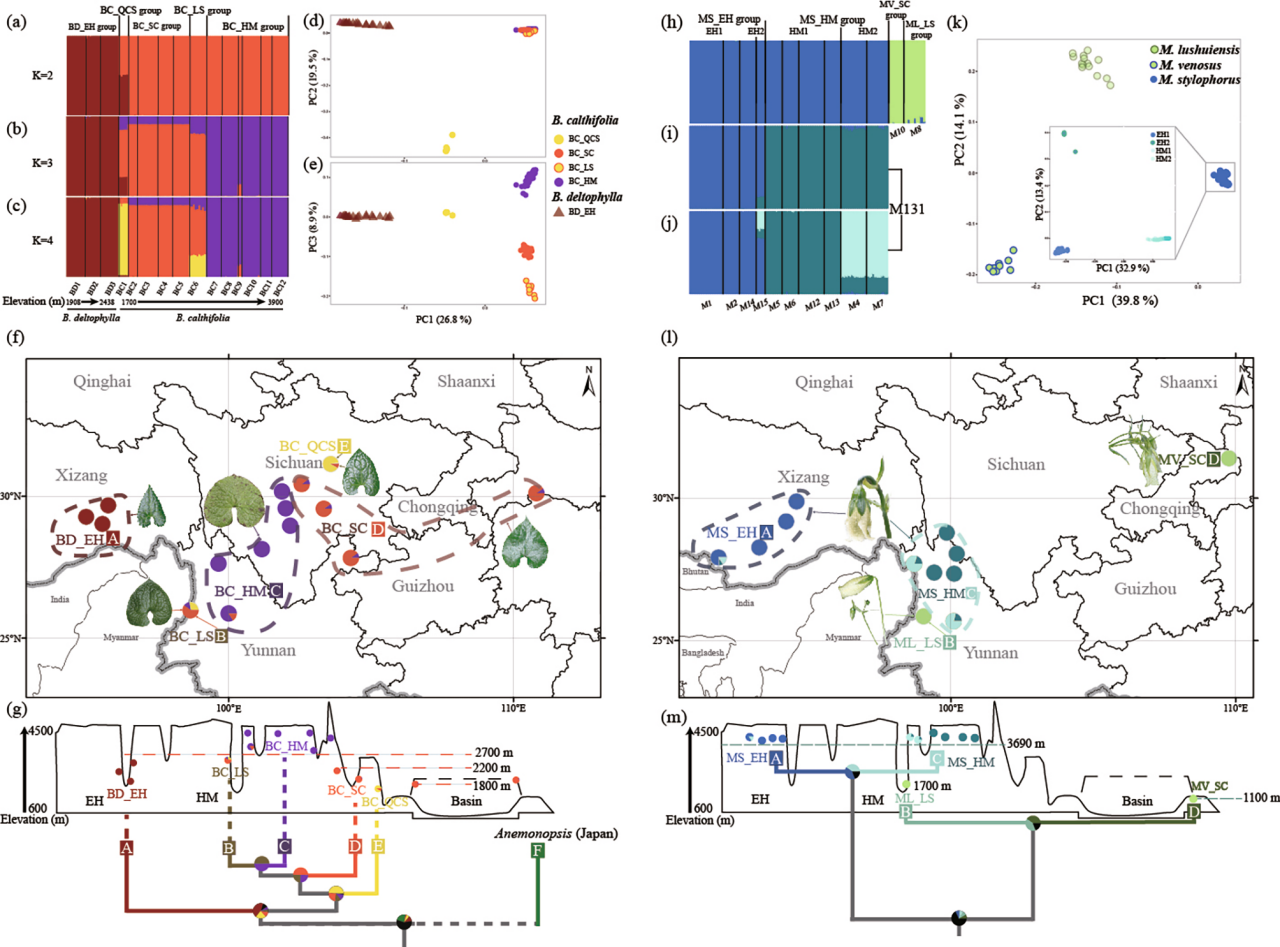
Population delimitation of *Beesia* (**a**-**g**) and *Megacodon* (**h-m**). (**a**-**c**) Genetic structure of *Beesia* based on, respectively, K = 2–4 for dataset B119. (**d**-**e**) PCA analysis based on dataset B119. (**f**), geographic distribution of genetic structure of *Beesia* based on K = 4. Dashed circles in (**f**) encompass groups inferred by STRUCTURE. A representative leaf from each *Beesia* group is shown. (**h**) Genetic structure of *Megacodon* based on K = 2 for dataset M155. (**i**) and (**j**) are genetic structure based on, respectively, K = 2 and K = 3 for dataset M131. (**k**) PCA analysis based on dataset M155 (outer) and M131 (inner). (**l**), geographic distribution of genetic structure of *Megacodon* based on K = 4 for dataset M155. A representative flower from each *Megacodon* species is shown. Dashed circles in (**l**) encompass groups of *M. stylophorus* inferred by STRUCTURE at K = 4. Schematic views of altitudinal distribution of genetic structure and phylogenetic relationships of *Beesia* (**g**) and *Megacodon* (**m**). Genetic structure for *Beesia* in (**g**) were inferred from B119 dataset. Orange dashed line and numbers in (**g**) show the elevation of BC_HM (> 2700 m) and BC_SC groups (1800–2200 m). Genetic structure for *Megacodon* in (m) were inferred from M155 datasets. Blue dashed lines and numbers in (**m**) show elevation of *M. stylophorus* (> 3690 m), *M. lushuiensis* (1700 m) and *M. venosus* (1100 m). Phylogenetic trees at bottom are simplified versions from BEAST results presented in Fig. 1. The estimated geographic regions of occurrence are estimated by BioGeoBEARS. Pie charts in (**g**) and (**m**) on nodes represent the relative probabilities of occurrence areas for the *Beesia* and *Megacodon* corresponding to (**f**) and (**l**) respectively. Reconstructions resulting in more than two possible regions are shown in dark gray. EH, East Himalayas; HM, Hengduan Mountains; Basin: Sichuan Basin; black dashed line: mountains around Sichuan Basin

On the basis of the results of STRUCTURE analysis, we divided the populations of *Beesia* into five groups (Fig. 2a–c and f). The result of the delta-K distribution showed that the best K value for *Beesia* was 2 based on the B119 dataset (Additional file 1: Fig. S7). Three populations of *Beesia deltophylla* formed one group (BD_EH group) consistently from K = 2 to K = 4. Population BC1 from 1700 m a.s.l was identified as being of admixed ancestry between *B. deltophylla* and *B. calthifolia* at K = 2 and 3 but it was identified as a unique evolutionary group (BC_QCS group) at K = 4 (Fig. 2a-c). The populations (BC2–BC5) from lower elevation (1800–2200 m a.s.l) and those (BC7–BC12) from higher elevation (2700–4000 m a.s.l) formed as two separate groups (BC_SC and BC_HM group) when K = 3 and 4 (Fig. 2g). Interestingly, samples collected from Lushui (population BC6) at 2660 m a.s.l were identified as of admixed ancestry (BC_LS group) between group BC_QCS and BC_SC at K = 4 (Fig. 2c and g). BC1 and BC6 contained the highest proportion of private alleles among the *Beesia* species (Additional file 2: Table S5). Furthermore, the BD_EH and BC_QCS groups were divergent from other populations according to the first two axes of the PCA plot, which explained 26.8% and 19.5% of the total variation (Fig. 2d). The third principal component (explaining 8.9%) showed substructure within the BC_SC, BC_LS, and BC_HM groups (Fig. 2e). The results were confirmed by phylogenetic inference (Additional file 1: Fig. S1).

The results of genetic diversity within populations of *Beesia* showed that the variation range of nucleotide diversity and observed heterozygosity of *B*. *deltophylla* (0.013 ≦ π ≦ 0.026, 0.019 ≦ *H*_obs_ ≦ 0.023) was lower than that of *B*. *calthifolia* (0.017 ≦ π ≦ 0.051, 0.017 ≦ *H*_obs_ ≦ 0.062; Additional file 2: Table S5). Among the *B*. *calthifolia*, the populations of BC_QCS (Private = 14.11%) and BC_LS (Private = 6.91%) contain higher proportions of private alleles than other populations (Additional file 2: Table S5).

Populations of *Megacodon* formed two clusters in STRUCTURE based on dataset M155. The result of the delta-K distribution showed that the best K value was 2 for both *Megacodon* and *M. stylophorus* based on the M155 and the M131 dataset respectively (Additional file 1: Fig. S8). *Megacodon venosus* and *M*. *lushuiensis*, from lower elevations (1100 and 1700 m respectively), formed one group (Fig. 2h and l), while *M. stylophorus* from higher elevations (3690–4000 m) formed another group (Fig. 2h). The STRUCTURE results based on dataset M131 showed that *M*. *stylophorus* could be divided into two major evolutionary clades (MS_EH and MS_HM) and four subclades (MS_EH1, MS_EH2, MS_HM1, and MS_HM2; Fig. 2i and j). The relationship was further confirmed by PCA and phylogenetic inference (Fig. 2k and Additional file 1: Fig. S2).

The results of genetic diversity within species of *Megacodon* showed that the nucleotide diversity of *M. stylophorus* (π = 0.101) was higher than that of *M. lushuiensis* (π = 0.084) and *M. venosus* (π = 0.051), while the observed heterozygosity of *M*. *stylophorus* (*H*_obs_ = 0.062) was lower than other two species based on dataset M155 (Additional file 2: Table S6). Among the *M*. *stylophorus*, all populations have similar nucleotide diversity and observed heterozygosity (Additional file 2: Table S7). The proportions of private alleles in *M*. *venosus*, *M*. *lushuiensis*, and MS_EH2 subclades were greater than 10% (Additional file 2: Tables S6 and S7).

### Historical range reconstruction and historical gene flow

The biogeographic model selection conducted through BioGeoBears using the BEAST tree and *Anemonopsis* from Japan as the outgroup, identified DIVALIKE + J as the most supported model, accounting for 58.17% of the relative weight (Additional file 2: Table S16). Biogeographical reconstructions showed that the common ancestor of *B*. *calthifolia* and *B*. *deltaphylla* was inferred to have established in the low elevations in Southwest China and the east Himalayan region (> 75%; Fig. 2g). Vicariance events likely played a significant role in the subsequent diversification and formation of each group of *Beesia* (Fig. 2g). High-level gene flows (*N*_m_ > 5) were found to only between BC_LS group and two widely distributed groups (i.e., BC_HM and BS_SC groups) as per the results of the MIGRATE-N analysis (Additional file 2: Table S12).

In the biogeographic analysis of *Megacodon* using the BEAST tree (excluding outgroups), the DIVALIKE + J model emerged as the predominant model with 41.98% relative weight (see Additional file 2: Table S17). The data showed that vicariance events from the East Himalayas to central China significantly influenced the diversification of *Megacodon* groups (Fig. 2m). The MIGRATE-N analyses showed that no significant historical gene flow among the three *Megacodon* species (Additional file 2: Table S13). Additionally, for *M. stylophorus*, high-level historical gene flows were only identified between MS_EH2 and other subgroups (Additional file 2: Table S14).

### Correlations between spatial genetic structure and geographic, elevation, and climatic factors

**Fig. 3:**
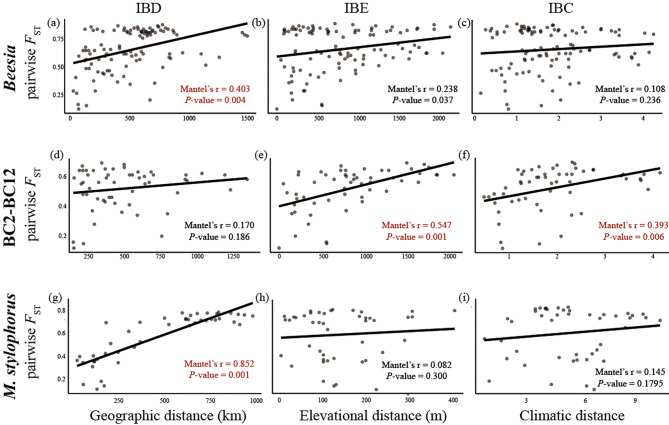
Correlations between pairwise genetic differentiation (pairwise *F*_ST_) based on B119 or M131 dataset and geographic (isolation-by-distance; **a**, **d**, and **g**), elevational (isolation-by-elevation, **b**, **e**, **h**), and climatic distance (isolation-by-climate, **c**, **f**, **i**). Numbers in the right lower corner are Mantel’s *r* and significance (*P*-value). The red font represents significantly strong correlation (Mantel’s *r* > 0.3 and *P*-value < 0.01)

Pairwise *F*_ST_ values between populations of *Beesia* ranged from 0.13 to 0.88 (Additional file 1: Fig. S3). The population BC1 showed the highest genetic differentiation from other populations of *B*. *calthifolia*, while BC6 had the second highest value (Additional file 1: Fig. S3). According to a Mantel test based on dataset B119, genetic differentiation showed significant correlation with geographic distance (isolation-by-distance, IBD, *r* = 0.403, *P*-value = 0.004) and elevational distance (isolation-by-elevation, IBE, *r* = 0.238, *P*-value = 0.037), but not climatic distance (isolation-by-climate, IBC, *r* = 0.108, *P*-value = 0.236, Fig. 3a-c, Additional file 1: Fig. S5). Partial Mantel tests further supported this result (Additional file 2: Table S8). After excluding *B. deltophylla* and the highly diverged population BC1, the genetic distances within BC_HM, BC_LS, and BC_SC groups increased significantly with elevational distance (*r* = 0.547, *P*-value = 0.001) and climatic distance (*r* = 0.393, *P*-value = 0.006 Fig. 3d-f, and Additional file 2: Table S10). However, climatic distance is not an independent variable but exhibits a partial matrix correlation with elevational distance (*r* = 0.213, *P*-value = 0.121, Additional file 2: Table S8).

Pairwise *F*_ST_ values between populations of *M. stylophorus* ranged from 0.11 to 0.78 (by dataset M131), indicating a high level of genomic differentiation in this species (Additional file 1: Fig. S4). The highest *F*_ST_ was found between M15 and other populations. There was a significant correlation between pairwise *F*_ST_ and geographic distance for all the populations (*r* = 0.86, P-value = 3e-05, Fig. 3g and Additional file 2: Table S8), while elevational (*r* = 0.082, *P*-value = 0.3, Fig. 3h) and climatic distance (*r* = 0.145, *P*-value = 0.1795, Fig. 3i) were not correlated with pairwise *F*_ST_.

### Adaptive and ecological divergence of *Beesia calthifolia* across elevation in the Hengduan Mountains

**Fig. 4:**
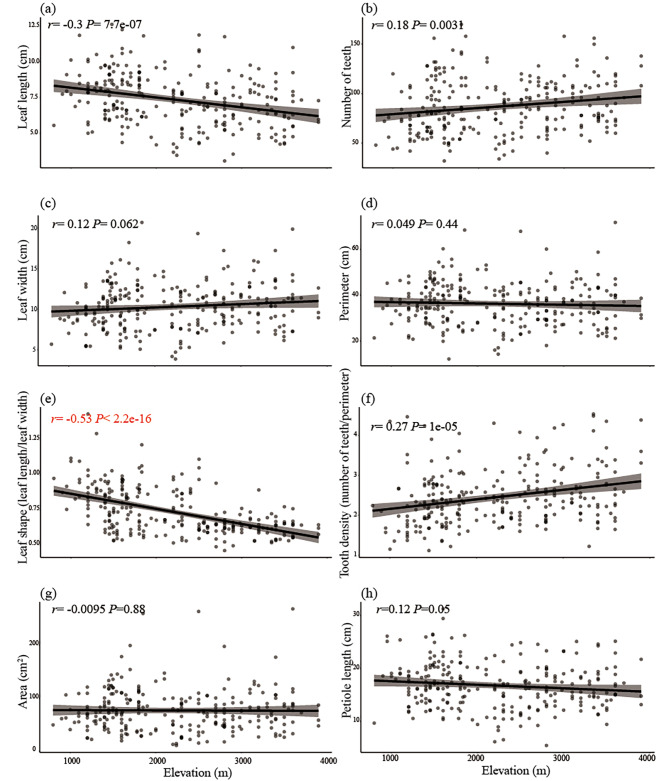
Linear regression analysis of correlation between leaf traits for *B*. *calthifolia* and elevation based on 258 specimens. (**a**): leaf length (cm), (**b**): number of teeth, (**c**): leaf width (cm), (**d**): perimeter (cm), (**e**): leaf shape (represented by ratio of leaf length to leaf width), (**f**): tooth density (calculated by number of teeth divided by perimeter), (**g**): leaf area (cm^2^), (**h**): petiole length (cm). The numbers in the left upper corner are correlation coefficient (*r*), with its significance (*P*-value), between each trait and elevation. The red font represents significantly strong correlation (|*r*| > 0.3 and *P*-value < 0.01)

Ecological divergence can lead to clinal variation, which in turn can result in genetic differentiation among populations. To further investigate the relationship between clinal variation and genetic differentiation, we first conducted a measurement on 258 specimens of *B*. *calthifolia*, which were collected at elevations ranging from 800 to 3900 m. Of these specimens, 79 individuals were collected at elevations higher than 2700 m, which is similar to the BC_HM group (Additional file 2: Table S15 and Additional file 3: Table S20). Our results indicate that leaf traits exhibit significant variability among all individuals, with coefficients of variation (CV) greater than 20% considered high (Additional file 2: Table S15). Among the eight traits studied, three traits (leaf length, leaf shape, and leaf teeth density) were found to differ significantly between high elevation (specimens ≥ 2700 m) and low elevation (specimens < 2700 m) specimens (Additional file 1: Fig. S9 and Additional file 2: Table S15). Additionally, three leaf traits (leaf length, leaf teeth density, and the number of teeth) were found to have a weak correlation with elevation (0.15 < |*r*| ≦ 0.3, *P*-value < 0.01, Fig. 4). However, the ratio of leaf length to width was found to have a strong negative correlation with elevation (*r* = -0.53, *P*-value < 2.2e-16, Fig. 4). Furthermore, elliptic Fourier descriptors and principal component analysis (EF-PCA) of 58 intact leaves revealed that the ratio of leaf length to width could explain 75.31% of the variation in leaf shape (PC1) (Additional file 1: Fig. S6). These findings suggest that leaf shape variation in *B. calthifolia* is significantly influenced by elevation, and that the ratio of leaf length to width is a key factor in this variation.

The aim of the ecological niche modeling (ENM) analysis is to understand whether *B*. *calthifolia* from two elevational ranges will respond differently to past climate change. The niche identity test was used to detect the niche divergence between *B*. *calthifolia* from two elevational ranges. All ENM models exhibit a strong predictive ability with area under the curve (AUC) exceeding 0.95. The predicted distribution range under current conditions matched the actual distributions of *B*. *calthifolia* occurring at both elevational ranges (Additional file 1: Fig. S10). The ENM results showed that the geographic ranges of both high- and low-elevation populations shrank during the LIG compared to their current ranges (Additional file 1: Fig. S10). The predicted distribution of high-elevation populations during the LGM were southward expansion (Additional file 1: Fig. S10). In contrast, the predicted distribution of low-elevation populations has tended to migrate to Sichuan Basin during the LGM (Additional file 1: Fig. S10). Discrepancies between the expected and observed values of Schoener’s *D* and Warren’s *I* suggest significant niche divergence between populations from the two elevational ranges (*p* < 0.01; Additional file 1: Fig. S11).

## Discussion

### Allopatric divergence in *Beesia* and *Megacodon*

Through reconstructing phylogenetic relationships and population structure in *Beesia*, we revealed an unexpectedly complex process of allopatric divergence in this genus. The genus most closely related to *Beesia* is *Anemonopsis*, which is endemic to Japan. However, the genetic divergence between *B. calthifolia* and *B. deltophylla* occurred at the western end of the total geographic range of *Beesia* around the late Miocene (Figs. 1b and 2g). Subsequently, the BC1 (Dujiangyan) population of *B. calthifolia* on the opposite side of the range diverged from other conspecific populations around the middle of the Pliocene (Fig. 2g). The result of BioGEOBears showed that all these divergences are the result of vicariance events, suggesting there may have been a widespread ancestral species for *Beesia* that existed at relatively lower elevations from the eastern Himalayas to central China (Fig. 2g). The Dujiangyan population was found to retain shared ancestral polymorphisms with *B*. *deltophylla*, as evidenced by K = 2 and 3 in our STRUCTURE analyses (Fig. 2a and b). A very small historical gene flow (*N*_m_) was detected between the Dujiangyan population and *B. deltophylla* and these two groups are isolated geographically, so the genetic similarity of these two groups may result from retention of ancestral polymorphisms of this widespread ancestral species at relatively lower elevations (Additional file 2: Table S12). In addition, population BC_LS (= 2640 m) was found to retain a greater proportion of shared ancestral polymorphisms with the group BC_SC (< 2200 m) than its adjacent populations in group BC_HM (> 2700 m) (Fig. 2b and c). The genetic similarity of these geographically isolated lineages may have resulted from ancient fragmentation of the habitat of a widespread *Beesia* ancestor at relatively lower elevations in the Hengduan Mountains.

A very similar distribution pattern was also found in allopatric divergence of two *Megacodon* species at lower elevations, *M. venosus*, and *M. lushuiensis*, formed in two isolated areas [30], but these two species still grow in similar habitat. These relict lineages isolated in different lowland regions by retaining the niche of their most recent common ancestor. These ancient allopatric lineages contain very large numbers of private alleles (Additional file 2: Table S5) due to limited gene flow. Therefore, genetic drift may have played an important role in their divergence processes.

Allopatric divergence also played an important role in speciation and/or divergence history of *Megacodon stylophorus* (Fig. 2m). This species diverged from the other two lowland *Megacodon* species in the late Miocene (6.28 Ma; Figs. 1d and 2h-m). Although *M. stylophorus* occupies a much higher elevation, this species has a distribution largely allopatric to those of the other two species and very limited historical gene flow was found between them, so the initial separation of this species may have resulted from allopatric divergence. After the initial divergence, significant intra-specific divergence in *M. stylophorus* was found between the Hengduan Mountains and the East Himalayas (Fig. 2h-m).

Because the same habitat type is highly fragmentized in the whole Sino-Himalayan region, relict lineages or species could be preserved in different isolated regions. We found that the allopatric divergence within these two genera occurred mainly within the same elevation range and among three separate sites within the Sino-Himalayan region: the mountain areas around the Sichuan Basin, the northwestern Yunnan (especially Gaoligong Mountain and its nearby areas), and the East Himalayas. These areas may all have stable humid climates in relatively low elevations in the last few million years because of the modern Asian monsoon system and their special geographical locations. These three regions are isolated from each other by peripheral high mountains, hot dry valleys, or regions that experience seasonal drought at lower elevations, which could have accelerated the allopatric divergence of many temperate taxa that favor humid climates [38, 39].

The historical fragmentation of humid region and the drastic geological changes (e.g., the landform formation of alpine valleys) in intermediate region may give rise to a phenomenon that early-diverged species (lineages) or sister species (lineages) were persistently isolated at opposite ends of the distribution area. The allopatric divergence in *Beesia* occurred successively at opposite ends of the distribution areas. The BC_HM group in the central distribution area is a young evolutionary lineage in *Beesia* and it occupies the highest elevations (Fig. 1b and 2g). *Megacodon stylophorus* also occurs in area between two *Megacodon* species at lower elevations and occupies the highest elevations in the genus. This pattern can also be found in the allopatric divergence of another East Asian endemic genus, *Rodgersia* [40], and an important tree group which favor humid habitat, *Tsuga* [41]. In these genera, the species (lineages) in the central region (e.g., several mountain chains in the Hengduan Mountains and the Yunnan Plateau) underwent adaptive changes because of the drastic geological and climatic changes [15], which may have created young species or lineages.

The drastic geological changes and vegetation evolution in the central region can also cause local extinction. Some temperate plant species or groups that favor humid habitats, such as *Davidia involucrata*, *Dickinsia hydrocotyloides, Dicentra macrantha*, and *Asteropyrum peltatum* [42], all have the same fragmented disjunction on Gaoligong Mountain or in nearby areas. However, the main distribution ranges of these species are all humid regions around the Sichuan Basin. This disjunction pattern in these species may be related to widespread areas of unsuitable habitat (e.g., pinewood) and seasonal drought in central Yunnan. The historical vegetation in central and west Yunnan may be closely related to the strength of the Asian monsoon [43]. When the Asian monsoon was weaker in late Pliocene, the humid habitats may become more fragmentized, resulting in local extinction or adaptive change of temperate taxa that favor humid climates.

Another conspicuous disjunction pattern within *Beesia* and *Megacodon* was found between the East Himalayas and the Hengduan mountains (between two *Beesia* species or within the species *M. stylophorus*). Many other temperate taxa, such as *Maddenia*, *Rodgersia* [40], and *Roscoea* [19], showed similar regional disjunctions or fragmented distributions. Interestingly, even some drought-tolerant species can also show a fragmented distribution between the East Himalayas and the Hengduan mountains [44]. We believe that an important biogeographic boundary once existed between the Nujiang and the Yarlung Zangbo River. This boundary may have been closely related to geological events in this region, such as the historical river capture of the Yarlung Tsangpo river [45, 46]. In addition, an ecological barrier between the Himalayas and the Hengduan Mountains was proposed in the study of *Roscoea* [19].

### Ecological divergence in *Beesia* and *Megacodon*

While ecological adaptation can play a role in the divergence of species over time, it may not be the primary cause of initial divergence processes. Instead, it may be a consequence that emerges after prolonged periods of allopatric divergence. But ecological divergence can reduce the effect of subsequent gene flow, whether under the circumstance of sympatry or secondary contact [47]. Although we have no evidence about ecological processes trigger incipient speciation or divergence directly in *Megacodon* and *Beesia*, ecological divergence indeed promotes subsequent lineage or species diversification in these two genera.

In our study, *M. stylophorus* and two lowland species occupy two distinctly different ecological niches and have fairly different morphological characters [30]. Although they are not sympatrically distributed, *M. stylophorus* and *M. lushuiensis* can co-occur in the Nushan Mountains. Habitat differentiation may be an important factor in the persistent divergence between *M. stylophorus* and the species at lower elevations. Our previous study found that many genes with functions in response to water deprivation and other external stimuli were under positive selection between *M. stylophorus* and *M. lushuiensis* [30]. According to the molecular dating analyses, the initial divergence of *Megacodon* took place in the late Miocene (Fig. 1d). This time coincides with a period when the East Asian monsoon was intense [48, 49] and there was heightened tectonic activity in the Hengduan Mountains [50–56]. This tectonic activity and the emergence of alpine valleys in the Hengduan Mountains made many lowland areas turn into hot dry valleys that were not suitable for the survival of *Megacodon* [57, 58]. At the same time, because monsoon intensification brings abundant moisture at high elevations, many species were able to colonize the high-elevation environment through adaptive evolution [15].

Habitat differentiation should play an important role in the persistent divergence of the BC_QCS group and the other groups of *Beesia* in the Hengduan mountains. The BC_QCS group and the populations in the group BC_SC can co-occur in the Qionglai mountains and can even be found in a very short distance, but they occupy different elevations and occur in different vegetation types. As we mentioned above, the BC_QCS population grows in relatively lower elevation and mainly grow in the understory of evergreen broad-leaf forest, which is similar to those of *B. deltophylla.* The other groups in the Hengduan mountians grow in the understory of deciduous or coniferous forests. Except for the BC_QCS group, the divergence of other three *B. calthifolia* groups was impacted by ecological divergence in altitudinal gradient, although *Beesia* showed an isolation-by-distance pattern across the whole distribution range (Fig. 4). We identified four groups in *Beesia calthifolia* based on STRUCTURE and PCA analysis (Fig. 2a-e). Of these, BC_HM, BC_LS, and BC_SC are three independent lineages based on all the datasets (Figs. 1 and 2 and Additional file 1: Fig. S1). The results of the Mantel test and partial Mantel test between genetic distance and climatic, geographic, or elevational distance showed that isolation-by-elevation was the main cause of genetic divergence among BC_HM, BC_LS, and BC_SC groups (Fig. 3e). We also found significant niche divergence between high and low elevation populations, as evidenced by niche identity test (Additional file 1: Fig. S11).

Changes in biotic and abiotic factors along altitudinal gradients can result in strong selective pressures and thus lead to clinal variation in morphological characteristics [59–62]. Leaves, being a key component of plant architecture and a medium for light capture, gas exchange, and thermoregulation [63], are particularly susceptible to such changes. Studies have shown that leaf shape and chloroplast evolution can contribute to the adaptation of plants to high elevations [63–65]. Our measurements of specimens revealed that leaf shape in different genetic lineages is strongly correlated with altitudinal gradients (Fig. 4e). Specifically, we found that groups at different elevations have distinct leaf shapes (Fig. 4e and Additional file 1: Fig. S6). Leaf traits, being classic functional characters that reflect changes along elevation or latitude gradients, are often directly linked to the local climatic environment [62, 66]. This relationship, as well as the underlying mechanisms, have been extensively studied in molecular biology and large-scale ecology research [67–69]. Therefore, the gradual changes in leaf shape across elevation gradients may be indicative of adaptation to varying environmental conditions. Additionally, as leaf shape is a plastic trait, we undertook a preliminary common garden transplant at the Kunming Institute of Botany to observe changes in leaf shape in the BC_HM, BC_LS, and BC_SC groups. We observed that the edges of individual leaves in the BC_HM group became concave when transplanted, while the leaves of the BC_SC and BC_LS groups grew normally. This suggests that leaf trait divergence in *B*. *calthifolia* is likely to be genetically based. However, for a more definitive conclusion, rigorous common garden or transplant experiments still need to corroborate this initial finding in the future.

According to molecular dating, the BC_HM group diverged from the BC_LS group in the early Pleistocene (Fig. 2). The Pleistocene era was marked by numerous glacial, which prompted the vertical migration of plants within mountainous regions [8, 58]. As a result, isolated populations of *B*. *calthifolia* may experience varying selection pressures during these glacial cycles. Meanwhile, the very different peripheral environments of the *B*. *calthifolia* clade at different elevations also led to a different response to the rapid climatic changes. From the result of ecological niche modelling, we found an apparent southward expansion of the distribution of the high-elevation clade during the LGM, while low-elevation populations tended to migrate to lower elevations (into the Sichuan Basin). This continuous isolation during glacial cycles may have further strengthened the divergence between high and low-elevation clade (Additional file 1: Fig. S10 and Fig. S11).

### The IBD + IBE synthesis effect in the Sino-Himalayan region

**Fig. 5:**
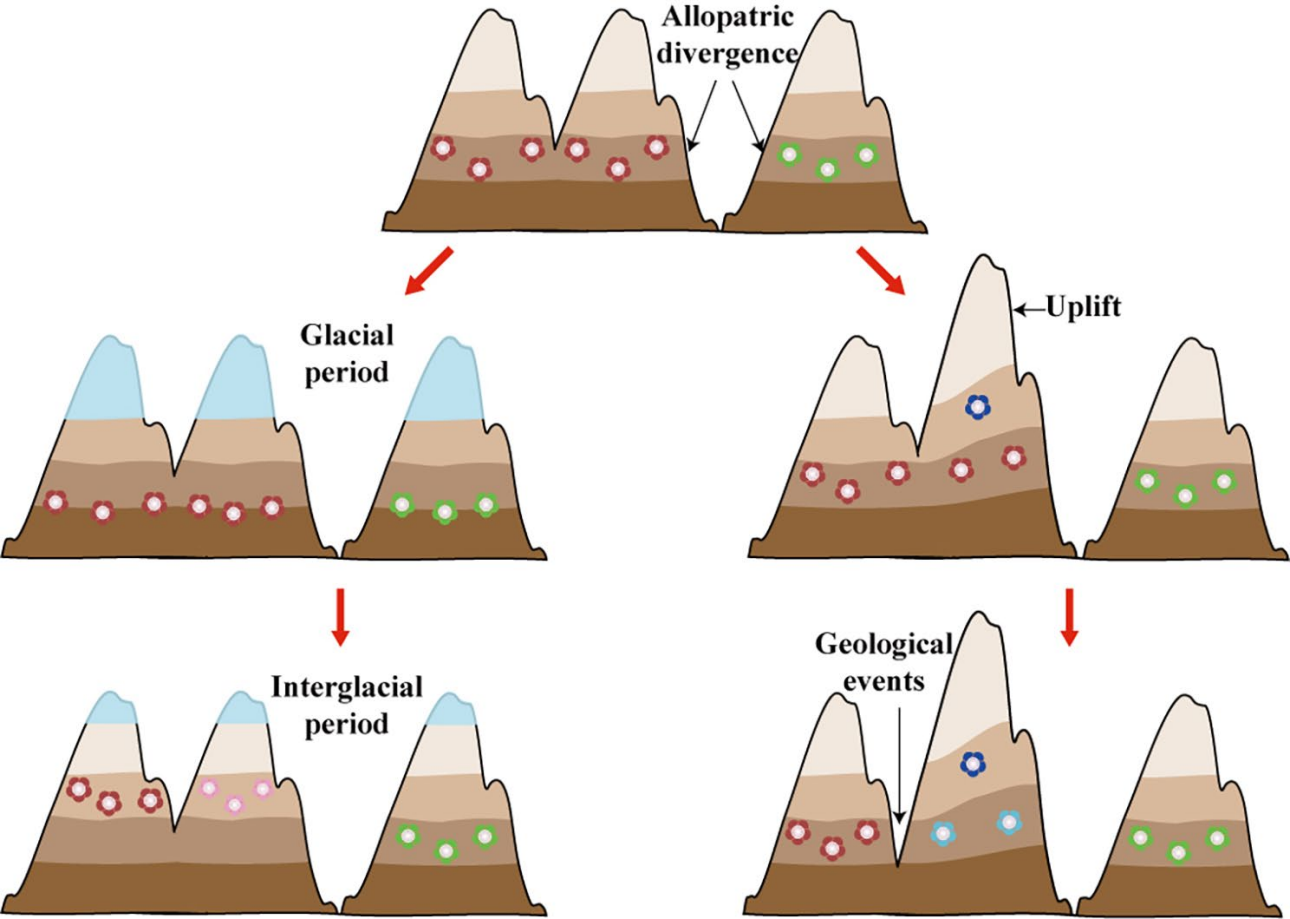
Schematic view of external factors influencing ecological divergence in Sino-Himalayan regions. The diagram at the top shows initial allopatric divergence. The two diagrams on the left show the vertical movement of plants caused by climate dynamics in the Pleistocene. The two on the right show tectonic activities and geological events including uplift, erosion, and historical river capture which can form new and isolated habitats leading to ecological divergence. Different color layers indicate elevation-related vegetation zones. Blue regions indicate ice sheets. Icons of small flower represent populations. Different colored icons represent different species

We found that the groups at higher elevations are largely allopatric with groups at relatively lower elevations in both *Beesia* and *Megacodon*. The loss of suitable habitat at the lower elevations may have forced these populations to migrate upward. Some populations probably colonized moist regions at high elevations during the interglacial period or the stage of the intensive Asian monsoon, but in areas where lower elevations are still suitable, relict populations may be preserved at lower elevations. A simultaneous allopatric and altitudinal isolation may have promoted speciation or intra-specific divergence of temperate plant taxa in the Sino-Himalayan region (Fig. 5), particularly those plant taxa with very specific habitat requirements. We believe that the complex divergence pattern observed in *Beesia* and *Megacodon* is applicable in many other temperate plant taxa in Sino-Himalayan region. However, the divergence process and geographical range evolution of a plant group are shaped by many factors. Different plant groups may have different life histories, habitat preference, demographics, and experience different tectonic and climatic events [70]. Even *Beesia* and *Megacodon* also have markedly different in their divergence processes, such as an earlier and sudden ecological divergence in *Megacodon*. Sometimes, the divergence events in some taxa may dominate by allopatric divergence because of strong niche conservatism, such as plant groups in extreme habitats. Furthermore, some taxa may involve more complex evolutionary histories, such as radiative diversification because of extensive hybridization [71], polyploidization or other unrevealed processes. Our study emphasizes the fact that habitat fragmentation and ecological adaptation at different elevations can work together in driving the divergence of plant groups in mountain regions. Plant groups in such regions are more likely to be isolated by environmental heterogeneity and physical barriers, resulting in habitat fragmentation and allopatric divergence (IBD). Different isolated populations may experience different selection pressures and also have different ecological opportunities, resulting in them colonizing new ecological niches (IBE). Sometimes, habitat fragmentation and ecological divergence interact. The effect of one process may be magnified by another. Different populations or groups in mountain regions can establish reproductive isolation quickly because of strong genetic drift and divergent selection. This IBD + IBE synthesis effect may be widespread as a factor leading to the divergence of plant groups in the Sino-Himalayan region.

## Conclusions

After conducting this comparative study between *Beesia* and *Megacodon*, it became evident that plant groups in mountain regions can diverge due to a combination of habitat fragmentation and ecological adaptation at different elevations. The presence of environmental heterogeneity and physical barriers in such regions can lead to isolation, resulting in habitat fragmentation and allopatric divergence. Furthermore, isolated populations can experience diverse selection pressures and opportunities, leading to the colonization of new ecological niches. These processes can interact and magnify each other, ultimately resulting in the establishment of reproductive isolation through strong genetic drift and divergent selection. Although the relative contributions of geographical isolation and parapatric ecological divergence vary among different plant taxa, a combination of these two factors is common in the Sino-Himalayan region.

## Methods

### Fieldwork and sampling

In order to study the population genetics of *Beesia* and *Megacodon*, a total of 119 samples were collected from 12 sites of *Beesia calthifolia* and three sites of *B*. *deltophylla*, as well as 10 individuals from one site of *M*. *venosus*, 14 individuals from one site of *M*. *lushuiensis*, and 131 individuals from 13 sites of *M*. *stylophorus* (Additional file 2: Table S1 and Table S2). The samples were collected to ensure that the distance between any two individuals sampled from each site was greater than 10 m. Leaf samples were collected and dried in silica gel for DNA extraction, and vouchers of each site were deposited at Herbarium of the Kunming Institute of Botany, Chinese Academy of Sciences (KUN). All samples were used for the population genetic analysis by ddRAD-seq (double digest restriction site associated DNA sequencing).

### Molecular dating based on molecular markers and chloroplast genomes

Since there is no prior information on the mutation rate for both genera before this study, we selected 16 individuals of *Beesia* and 16 individuals of *Megacodon* and combined previous published data for molecular dating analysis. To estimate divergence times among different populations or species, we used a combination of fossil records and secondary calibration. For *Megacodon*, the dataset used for molecular dating was obtained from [29] and [30], which used the nuclear ribosomal internal transcribed spacer (nrITS) and two chloroplast markers, *atpB*-*rbcL*, and *trnL-trnF*.

For *Beesia*, thirteen individuals from ten populations of *B*. *calthifolia* and three individuals from three populations of *B*. *deltophylla* were used to confirm phylogenetic relationships. We assembled chloroplast genomes for these *Beesia* samples and extracted protein-coding gene sequences (CDSs) for phylogenetic analysis and molecular dating, following the methods outlined in [35]. Paired-end 150-bp libraries were constructed and sequencing for *Beesia* was carried out on an Illumina HiSeq 2000 platform, and approximately 3 Gb data for each sample was generated. After filtering out low-quality reads, the GetOrganelle v1.7.1 pipeline [72] was used to assemble the whole chloroplast genome sequence. In this pipeline, plastid genome reads were extracted from total sequencing data and assembled with SPAdes version 3.10 [73]. The kmer values were set as 127, 115, 105, and 85. The sequences were further annotated in the PGA software package [74] using a published whole chloroplast genome from *Beesia calthifolia* (MK253467) [35] as reference and then checked manually in Geneious 8.2.4 [75]. We also downloaded chloroplast sequences of the Cimicifugeae from [35] as outgroups (Additional file 2: Table S9). Geneious 8.2.4 was then used for extraction of chloroplast CDSs. Alignment of matrix was performed by using MAFFT v 7.308 [76].

BEAST v1.84 [77] was used for molecular dating analysis. The secondary calibration setting for *Beesia* followed the results of [35], constraining the crown age of the Cimicifugeae (26.57 Ma) and the stem age of *Beesia* and *Anemonopsis* (6.4 Ma). The best-fit model of nucleotide substitution was GTR + I + R, which was estimated using jMODELTEST v 2.1.7 [78]. For *Megacodon*, the fossil calibration settings followed Favre et al. (2016) [29], constraining the stem age of *Lisianthius* (35.21 Ma) and the crown age of the *Gentiana* sect. Cruciata (6.25 Ma). The best-fit model of nucleotide substitution in BEAST analysis of *Megacodon* was the same as that given by [30]. A birth-death incomplete sampling tree prior was used, and a burn-in of the initial 10% cycles was carried out in BEAST. The analysis was run for 10^8^ Markov Chain Monte Carlo (MCMC) steps. MCMC samples were imported into TRACER v1.5 (available from http://beast.bio.ed.ac.uk/Tracer) to inspect the sampling adequacy and convergence of the chains to a stationary distribution. The results were visualized and plotted in FIGTREE v1.3.1 (available from http://tree.bio.ed.ac.uk/software/figtree/).

### Population genetics analysis based on RAD-seq

Genomic DNA was extracted using a Plant Genomic DNA kit (Tiangen Biotech, Beijing, China) following the kit protocol. Restriction site-associated DNA library preparation followed a universal and simplified ddRAD library method for angiosperm plants named MiddRAD [79]. Total genomic DNA (20 ng) was digested by MspI (New England Biolabs, NEB, #R0106L) and AvaII (New England Biolabs, NEB, #R0153L) at 37 ℃ for 3 h in a 20 µl reaction volume buffered with 1x CutSmart buffer (New England Biolabs, NEB, #B7204S). A fragment library with inserts of 400–600 bp was constructed strictly according to the protocol for MiddRAD. The Illumina HiSeq X Ten (San Diego, CA, USA) sequencing platform (PE150) was used for sequencing at Novogene Co. (Beijing, China).

Filtered clean reads were processed by the Stacks 2.0 Beta 8 software pipeline [80–82]. First, reads were demultiplexed by P1 barcode and low-quality reads were removed (-c, -q, -r). Then Fastp [83] was used to trim the low-quality bases. The first reads were trimmed by 11 bp at the 3’ end and 10 bp at the 5’ end, and the second reads were trimmed by 20 bp at the 3’ end and 10 bp at the 5’ end. Then these trimmed reads were assembled using denovo_map.pl. The parameter m represents the minimum number of reads to create a stack; the *M* represents the maximum number distance allowed between stacks; the *n* represents the maximum number of mismatches allowed between loci. The various parameter combinations by systematically varying *M* and *n* from 1 to 6 (*M* = *n*) and setting m at a constant value of 3 were explored. The values maximized the number of polymorphic loci found in 80% of the individuals were used (parameter settings: *m* = 3, *M* = 4, *n* = 4 for *Megacodon*; *m* = 3, *M* = 5, *n* = 5 for *Beesia*). The datasets for downstream population genetic analyses were generated by the POPULATIONS module and PLINK v.1.07 [84]. Each RAD locus presents in at least 50% of the individuals (-r 0.5) of each population and that appeared each population (-p 15 for dataset B119, -p 12 for dataset M155, -p 10 for dataset M131) was retained, and for each locus, unlinked SNP were retained (--write_single_snp). Finally, we used PLINK to filter out loci with minor allele frequency < 0.01and missing genotypes > 0.3 (--maf 0.01 –geno 0.3). Input files were converted by the POPULATIONS program implemented in Stacks and PGDSpider [85] for further analysis.

We used STRUCTURE 2.3.4 [86] to detect the underlying population structure among samples. Ten independent runs of each K-value in the range from 1 to 10 were performed with 5 x 10^5^ burn-in lengths and 10^6^ iterations. STRUCTURE HARVESTER [87] was performed to choose the optimal K-values by the delta-K method. We averaged the coefficients over the ten independent runs by CLUMPP version 1.1 [88] and plotted the output with DISTRUCT [89]. Principal component analysis (PCA) was performed by the Bioconductor package SNPRELATE [90]. Phylogenies were reconstructed using a maximum likelihood approach in RAxML 8.2.12 [91] with 1000 bootstrap replicates. The substitution model of GTR + GAMMA model with acquisition bias correction (--asc-corr = lewis) were assigned. Pairwise *F*_ST_ and genetic diversity (including private sites; observed heterozygosity, *H*obs; nucleotide diversity, π; *F*_IS_) were estimated by the POPULATIONS program in Stacks.

We used the MIGRATE-N 3.6.11 software to estimate the historical gene flow (*N*_m_) between populations and species [92]. This software utilizes a Bayesian inference strategy to infer the migration rates and divergence times of populations based on genetic data. For our analysis, we used three SNP datasets and set the following parameters: 500,000 visited parameter values (recorded steps = 5000, increment = 100, number of concurrent chains = 1), with a burn-in period of 10,000.

### Biogeography analysis

Ancestral range reconstructions for *Beesia* and *Megacodon* were conducted using BioGeoBEARS [93]. We employed three analyses: dispersal-extinction-cladogenesis (DEC), a likelihood version of BayArea (BAYAREALIKE), and a likelihood version of dispersal-vicariance analysis (DIVALIKE). Each analysis was performed both with and without the J-parameter, utilizing the time tree obtained from the aforementioned BEAST analysis. For *Megacodon*, outgroups were excluded due to the uncertainty of the *Megacodon*’s outgroup. *Anemonopsis* was retained because this genus is most closely related to *Beesia*. We defined four biogeographic regions based on the current distribution of *Megacodon*: (A) East Himalayas; (B) Lushui in Nushan; (C) Hengduan Mountains; and (D) Central China. In addition to these four regions, two more biogeographic areas were defined for *Beesia* and *Anemonopsis*: (E) Dujiangyan near the Sichuan Basin; and (F) Japan. The best-fit biogeographical model was determined using the AIC criterion.

### Isolation by distance (IBD), isolation by elevation (IBE), and isolation by climatic distance (IBC) analyses

To investigate the drivers of population genetic differentiation for *Beesia* and *Megacodon*, we conducted a Mantel test between genetic distance (represented by pairwise *F*_ST_) and climatic, elevational, or geographic distance using the function *mantel* of *vegan* [94] in R [95]. We also conducted a partial Mantel test, controlling these distance matrixes using the Pearson coefficient. The permutations were all set as 99,999. The geographic distance was generated by Geographic Distance Matrix Generator v. 1.2.3 (https://biodiversityinformatics.amnh.org/open_source/gdmg/index.php) based on the GPS reading at each sampling site. The climatic distance was calculated based on the Euclidean distance of the first two PCs from PCA analysis of 19 bioclimatic factors, which were extracted from the WorldClim database (https://www.worldclim.com/) by the raster package in R. The elevational distance was represented by the absolute value of elevation difference between every pair of populations.

### Ecological niche modelling and niche identity test of *Beesia calthifolia*

We classified our collection and the herbarium records of *B*. *calthifolia* into two groups based on an elevation threshold. The group has a high elevation distribution (above 2700 m) correspond to the BC_HM group in our study, while the group distributed at relatively lower elevation correspond to the BC_SC group, the BC_LS group and the BC_QCS group. After eliminating overlapping sites, we identified 16 habitat locations in the high-elevation group and 26 in the low-elevation group.

We estimated the potential distribution ranges of *B*. *calthifolia* during the LIG, the LGM, and the present using the bioclimatic variables from WorldClim. To mitigate the effects of collinearity, we excluded bioclimatic variables that had a Pearson’s correlation coefficient of *r* ≥ 0.85 [96]. Upon exclusion of bioclimatic variables with high Pearson correlation, eight variables were retained for the high-elevation group (i.e., BIO1, BIO2, BIO3, BIO7, BIO12, BIO14, and BIO19; Additional file 2: Table S18) and six for the low-elevation group (i.e., BIO1, BIO2, BIO4, BIO12, BIO13, and BIO15; Additional file 2: Table S19). We employed MaxEnt 3.4.4 [97] for distribution estimation, using default settings and specific parameters: random test (25%), training (75%), regularization multiplier (1), maximum iterations (5000), convergence threshold (0.00001), maximum background points (10,000), and 10 bootstrap replications. Model accuracy was gauged using the AUC of receiver operating characteristic (ROC) curve. Outputs were reclassified for visualization, with high suitability set at > 0.4.

For niche identity test of *Beesia calthifolia* distribution at different elevations, we used Schoener’s *D* [98] and Warren’s *I* [99] indices, which range from 0 (no overlap) to 1 (equal suitability). Using ENMTools v.1.4.4 [100], a niche identity test with 100 pseudo-replicates provided expected index values. We applied a nonparametric Monte Carlo test to compare observed and expected indices, with *p* < 0.01 indicating non-identical environmental niches between regions.

### Morphometrics of *Beesia calthifolia*

We downloaded specimen images of *Beesia* with elevation information from CVH (http://www.cvh.ac.cn/) and measured phenotypes using ImageJ. We then measured the 259 largest complete leaves (leaf width, leaf length, petiole length, leaf area, and leaf perimeter) from each specimen image. Statistical analysis was carried out using R version 3.5.2 [95].

In order to examine the effects of elevation on the principal component scores of leaf shape among the varieties, EF-PCA was performed by SHAPE software [101] which includes ChainCoder, Chc2Nef, PrinComp, and PrinPrint programs. Fifty-eight intact leaf images of specimens from different elevations were selected to convert into bitmap format using Windows Paint. Then we followed the SHAPE manual. We selected normalization based on the longest radius for normalizing the shape manually in Chc2Nef. The leaf shape was approximated by PrinComp and PrinPrint from the first 20 harmonics of the coefficients. The PCA was implemented in R [95].

### Electronic supplementary material

Below is the link to the electronic supplementary material.

Supplementary Material 1

Supplementary Material 2

Supplementary Material 3

## Data Availability

All genomic data are available from the National Genomics Data Center (https://ngdc.cncb.ac.cn/) under the accession number PRJCA007438. For any inquiries regarding the data, please contact Junchu Peng at junchupeng@163.com.

## Abbreviations

ddRAD-seq: Double digest restriction site associated DNA sequencing
PCA: Principal component analysis
π: Nucleotide diversity
*F*_IS_: Inbreeding coefcient
nrITS: Nuclear ribosomal internal transcribed spacer
CDS: Protein-coding gene sequence
MCMC: Markov Chain Monte Carlo
*F*_ST_: Genetic differentiation
*H*_obs_: Observed heterozygosity
*N*_m_: Historical gene flow
IBD: Isolation-by-distance
IBE: Isolation-by-elevation
IBC: Isolation-by-climate
LGM: Last Glacial Maximum
LIG: Last Interglacial
CV: Coefficients of variation
EF-PCA: Elliptic Fourier descriptors and principal component analysis
WCG: Whole chloroplast genome
