# Supplementary material for: Ancient allopatry and ecological divergence act together to promote plant diversity in mountainous regions: evidence from comparative phylogeography of two genera in the Sino-Himalayan region
Source: BMC Plant Biol. 2023 Nov 17;23:572. doi: 10.1186/s12870-023-04593-1 (PMC10655281; doi:10.1186/s12870-023-04593-1)
Supplement: Supplementary file 1 — Supplementary Material 1 [file 12870_2023_4593_MOESM1_ESM.pptx]

## Slide 1
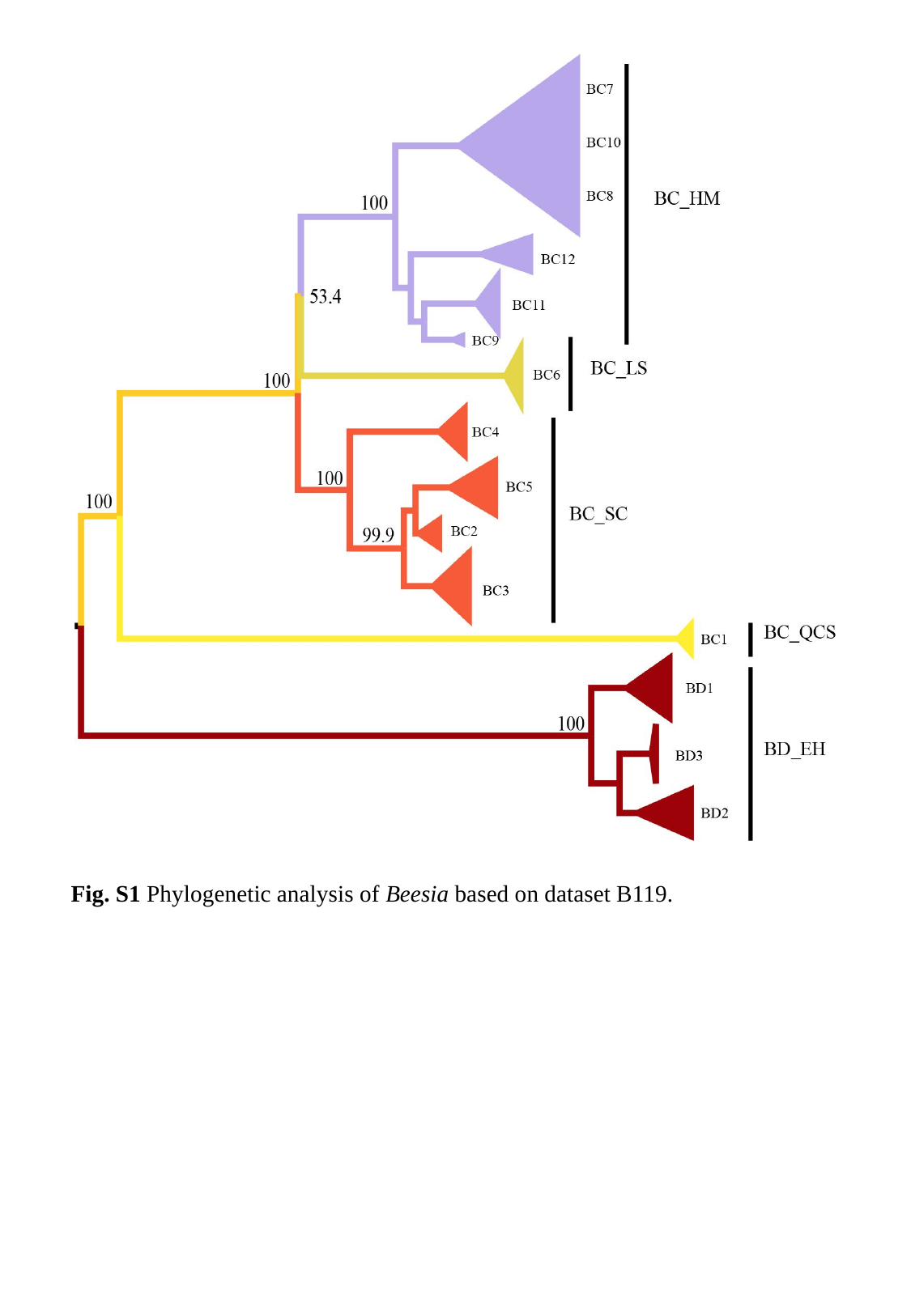

Fig. S1 Phylogenetic analysis of Beesia based on dataset B119.

## Slide 2
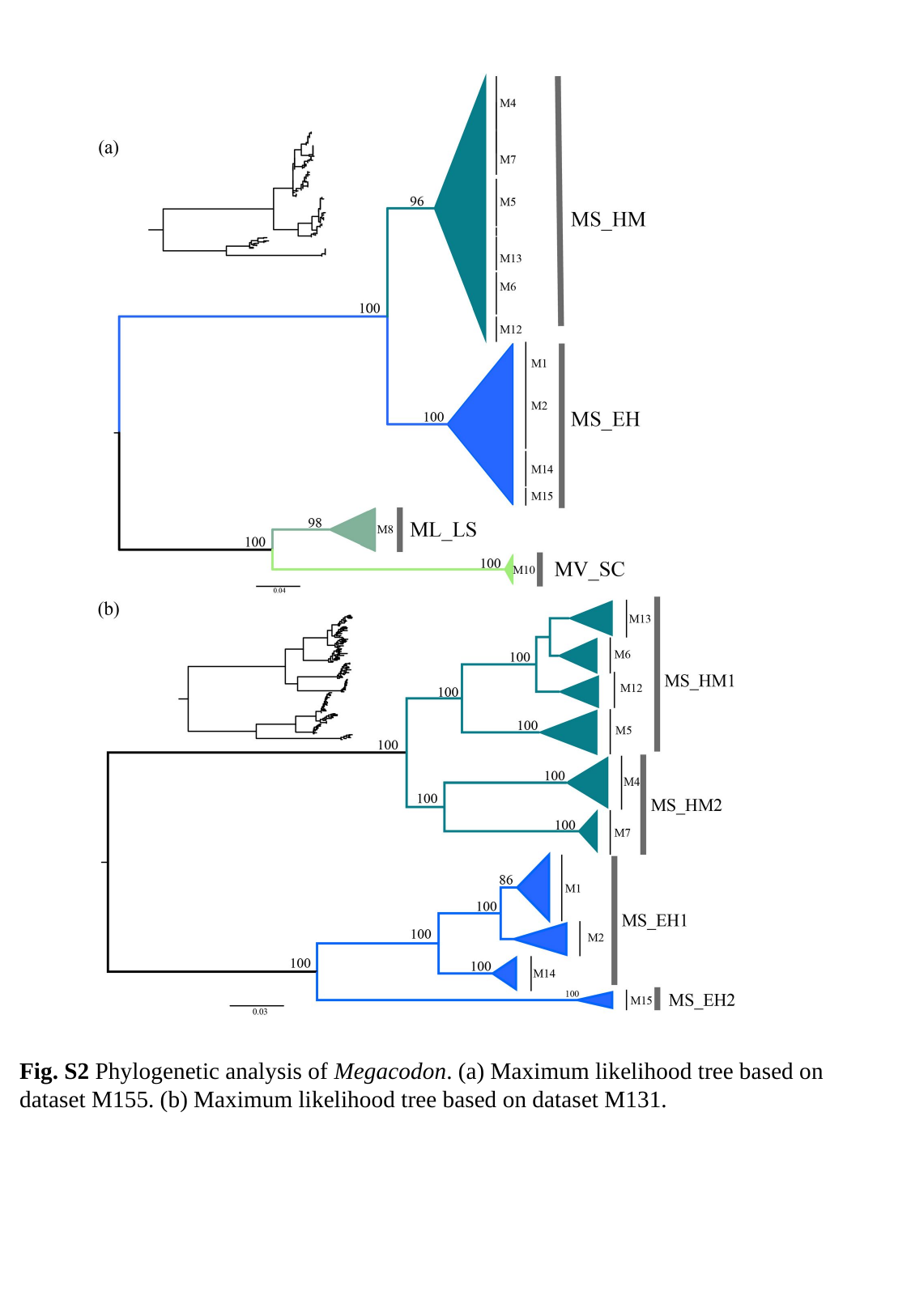

Fig. S2 Phylogenetic analysis of Megacodon. (a) Maximum likelihood tree based on dataset M155. (b) Maximum likelihood tree based on dataset M131.

## Slide 3
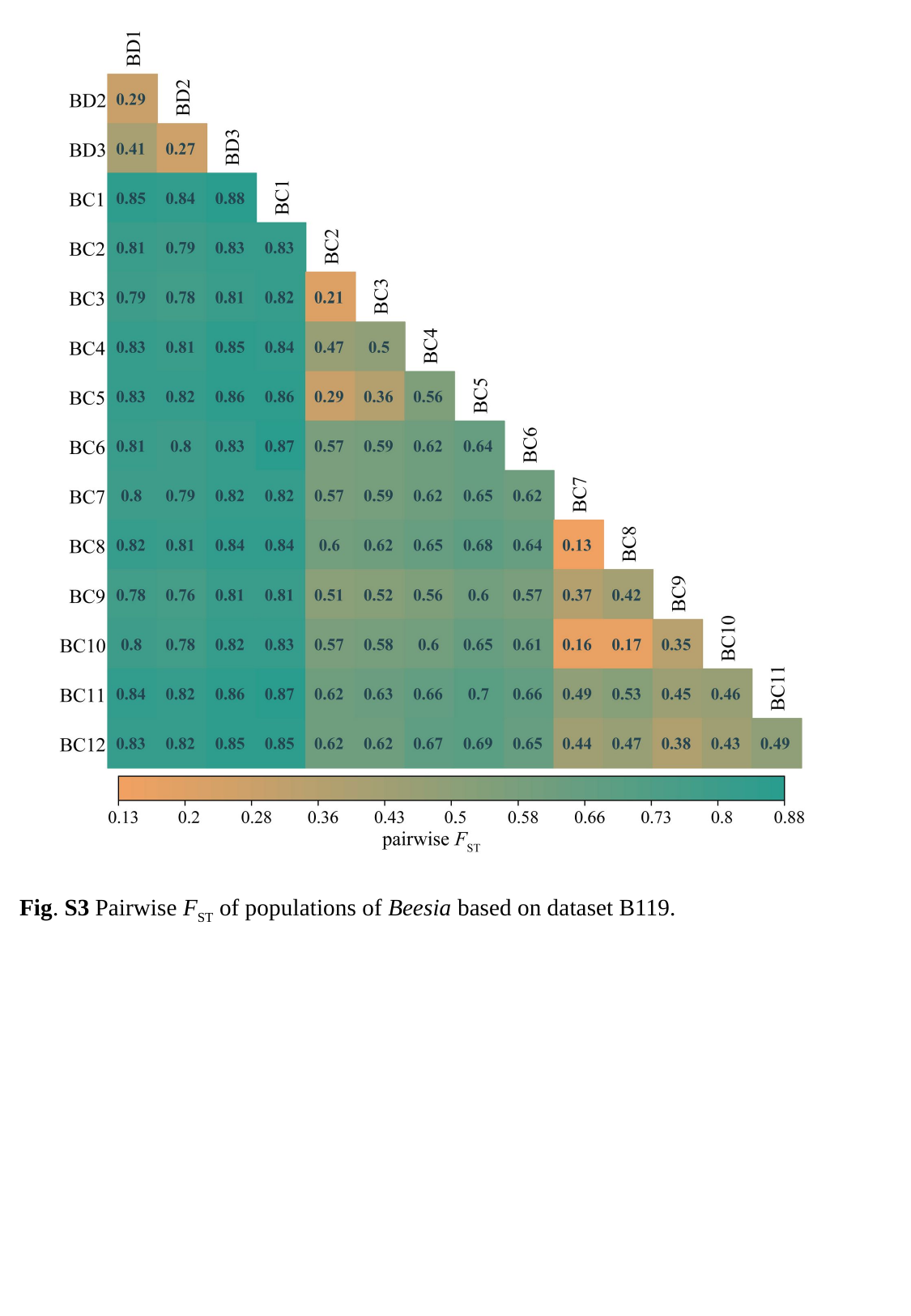

Fig. S3 Pairwise FST of populations of Beesia based on dataset B119.

## Slide 4
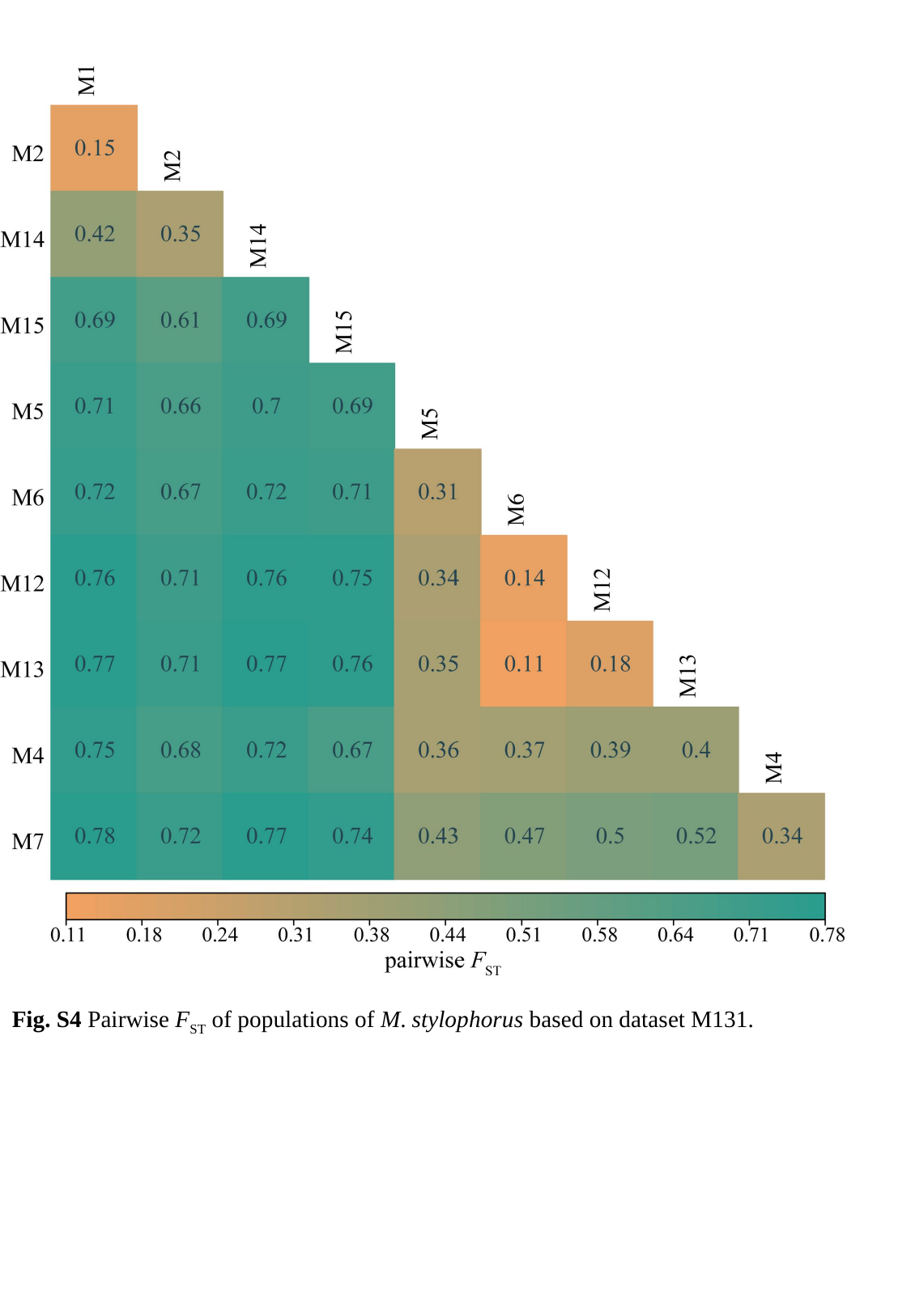

Fig. S4 Pairwise FST of populations of M. stylophorus based on dataset M131.

## Slide 5
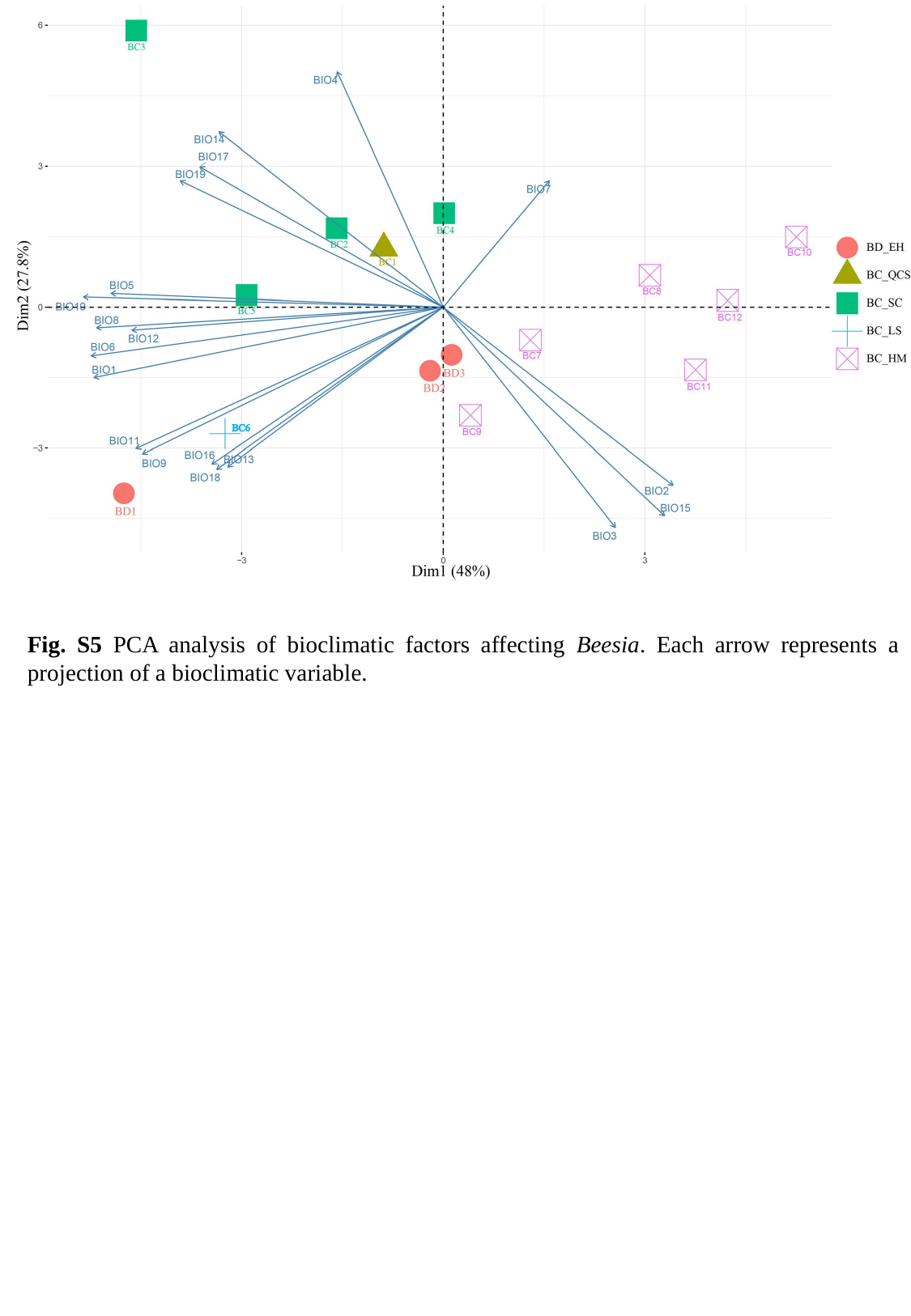

Fig. S5 PCA analysis of bioclimatic factors affecting Beesia. Each arrow represents a projection of a bioclimatic variable.

## Slide 6
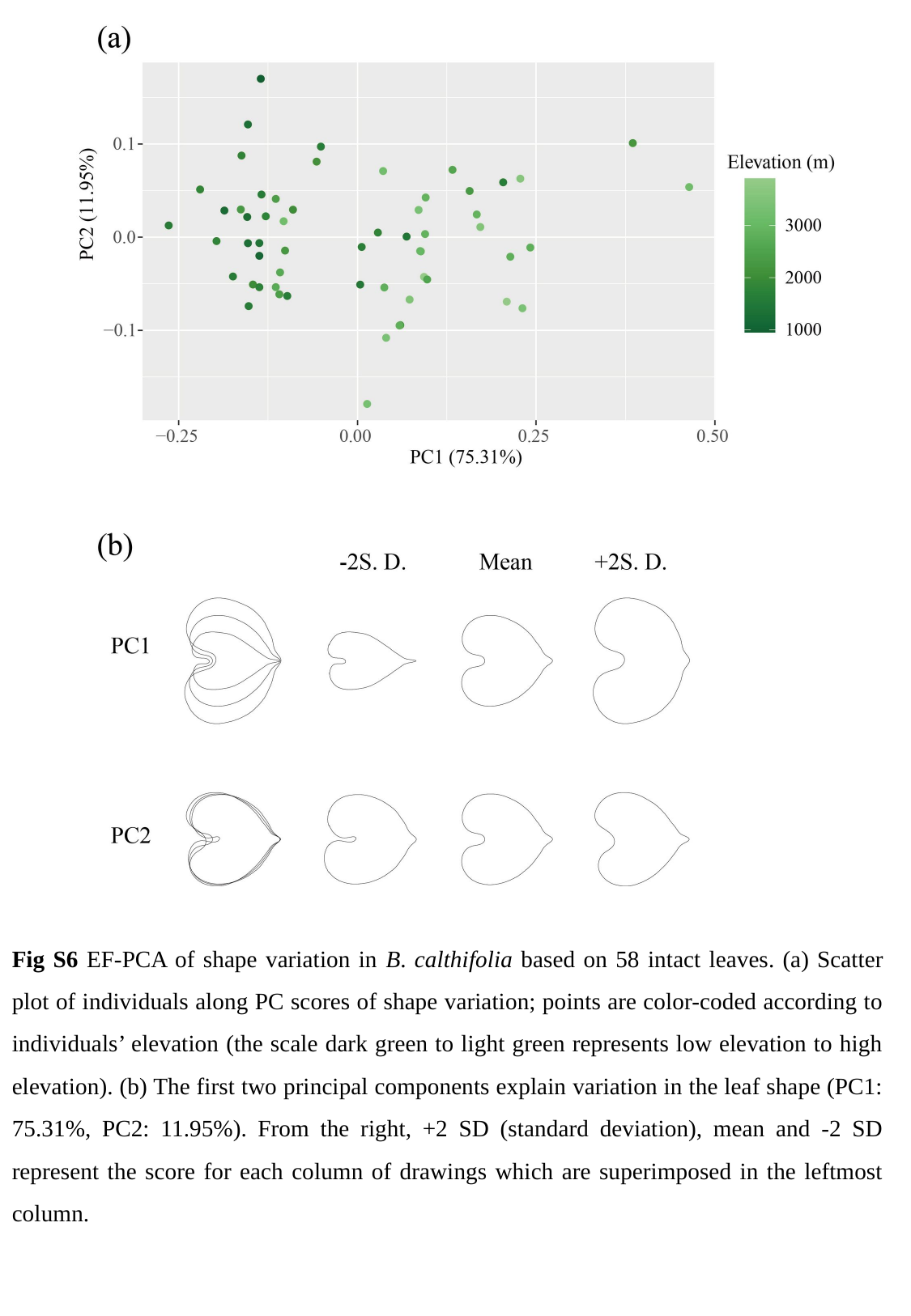

Fig S6 EF-PCA of shape variation in B. calthifolia based on 58 intact leaves. (a) Scatter plot of individuals along PC scores of shape variation; points are color-coded according to individuals’ elevation (the scale dark green to light green represents low elevation to high elevation). (b) The first two principal components explain variation in the leaf shape (PC1: 75.31%, PC2: 11.95%). From the right, +2 SD (standard deviation), mean and -2 SD represent the score for each column of drawings which are superimposed in the leftmost column.

## Slide 7
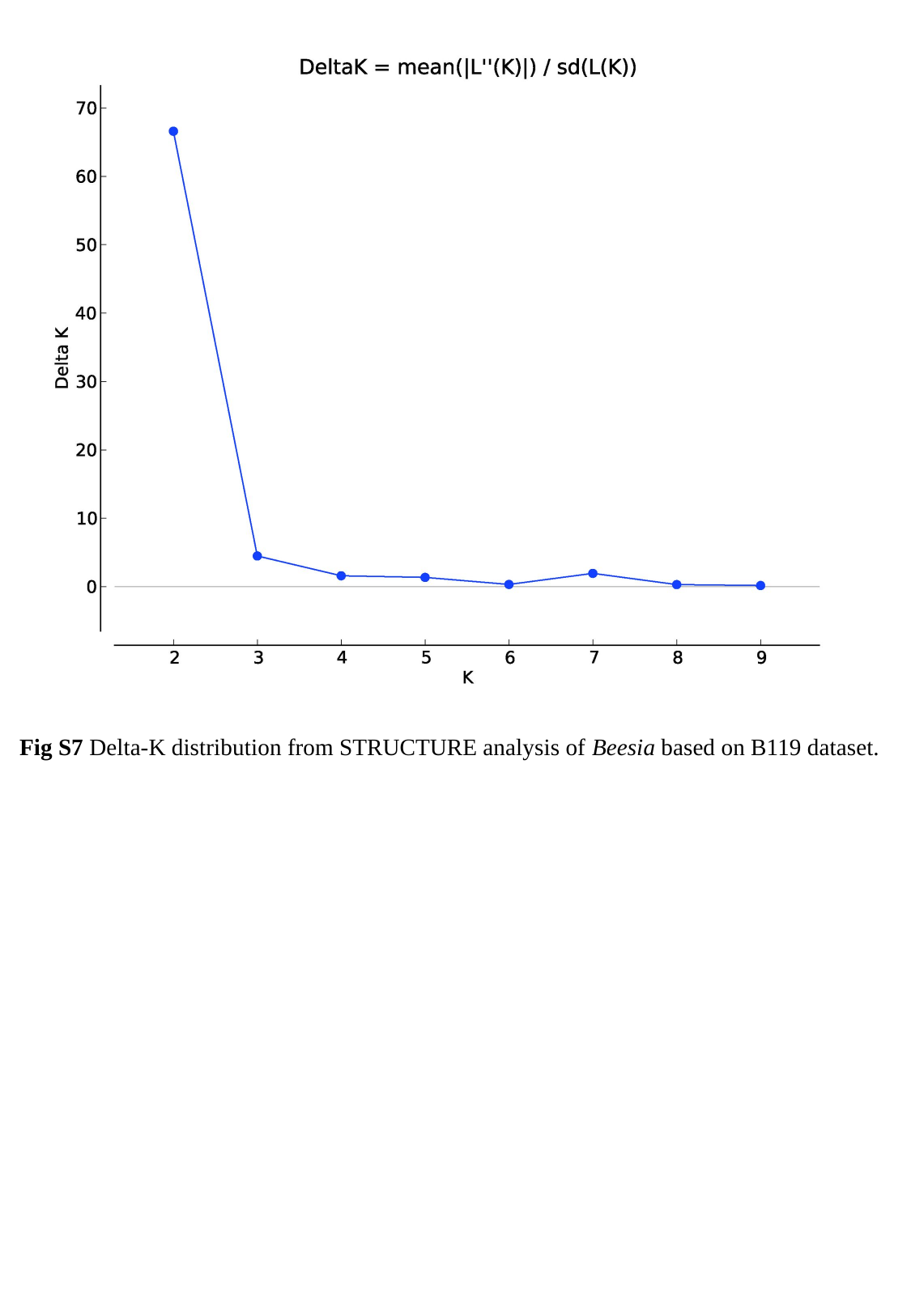

Fig S7 Delta-K distribution from STRUCTURE analysis of Beesia based on B119 dataset.

## Slide 8
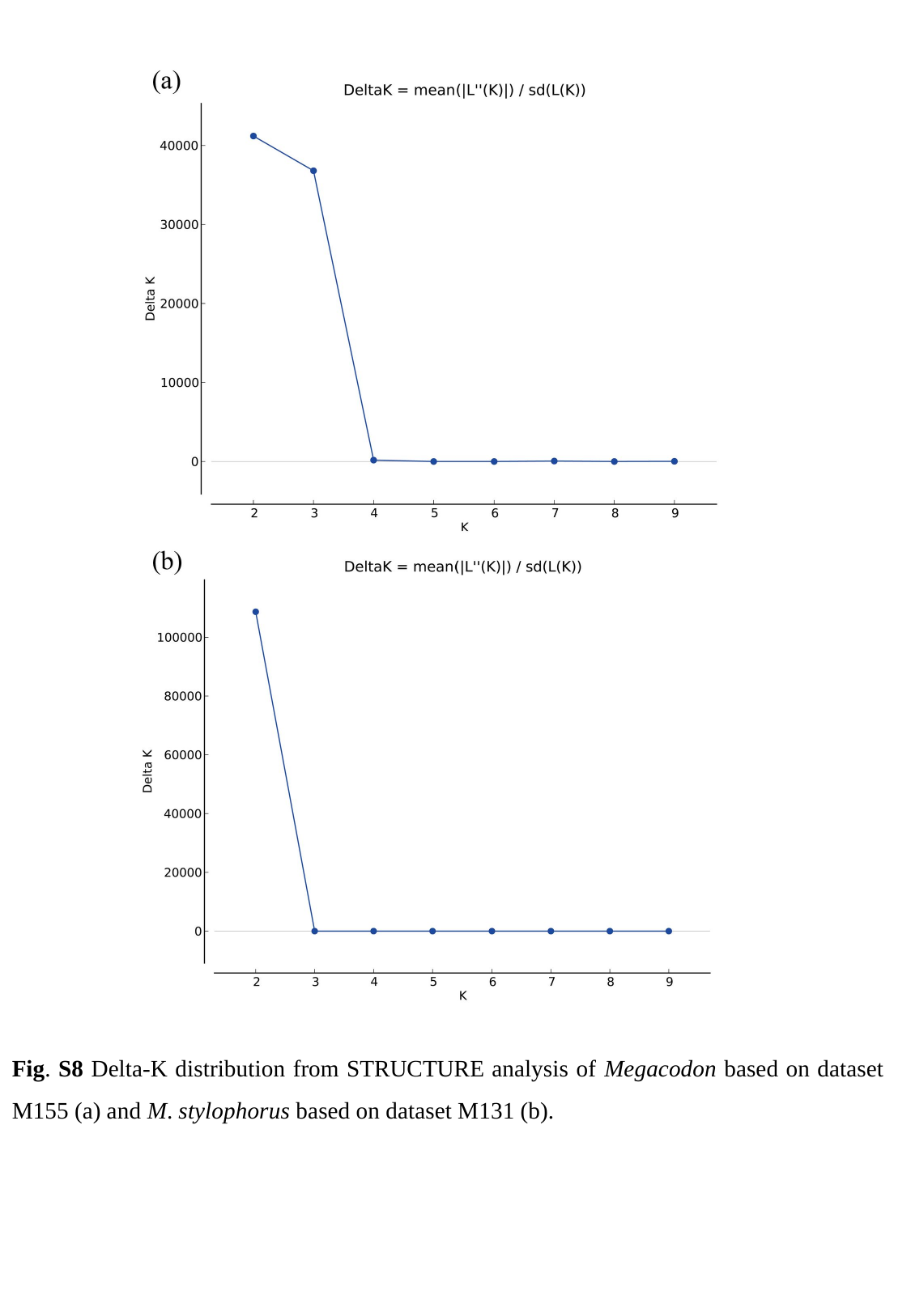

Fig. S8 Delta-K distribution from STRUCTURE analysis of Megacodon based on dataset M155 (a) and M. stylophorus based on dataset M131 (b).

## Slide 9
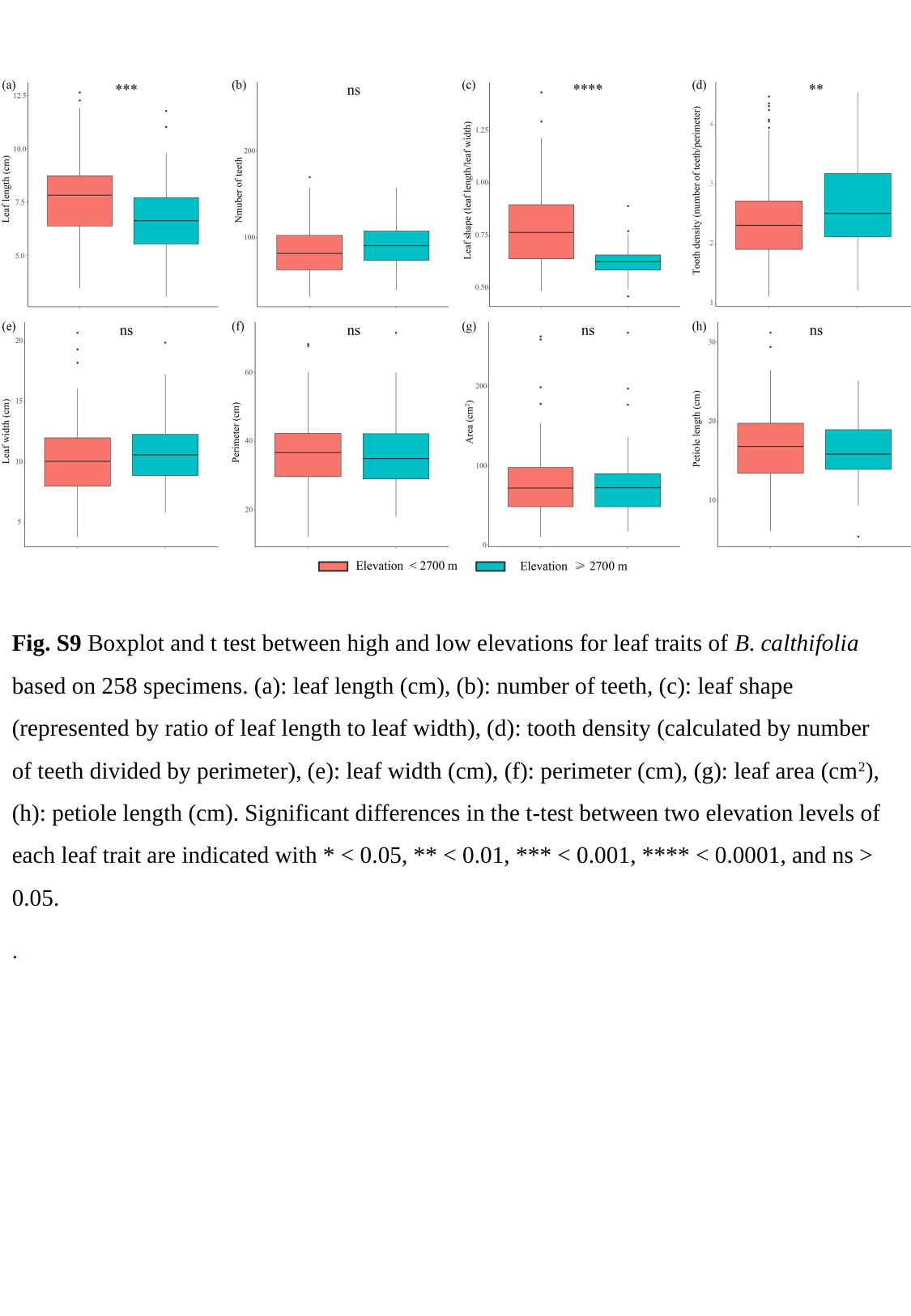

Fig. S9 Boxplot and t test between high and low elevations for leaf traits of B. calthifolia based on 258 specimens. (a): leaf length (cm), (b): number of teeth, (c): leaf shape (represented by ratio of leaf length to leaf width), (d): tooth density (calculated by number of teeth divided by perimeter), (e): leaf width (cm), (f): perimeter (cm), (g): leaf area (cm2), (h): petiole length (cm). Significant differences in the t-test between two elevation levels of each leaf trait are indicated with * < 0.05, ** < 0.01, *** < 0.001, **** < 0.0001, and ns > 0.05.
.

## Slide 10
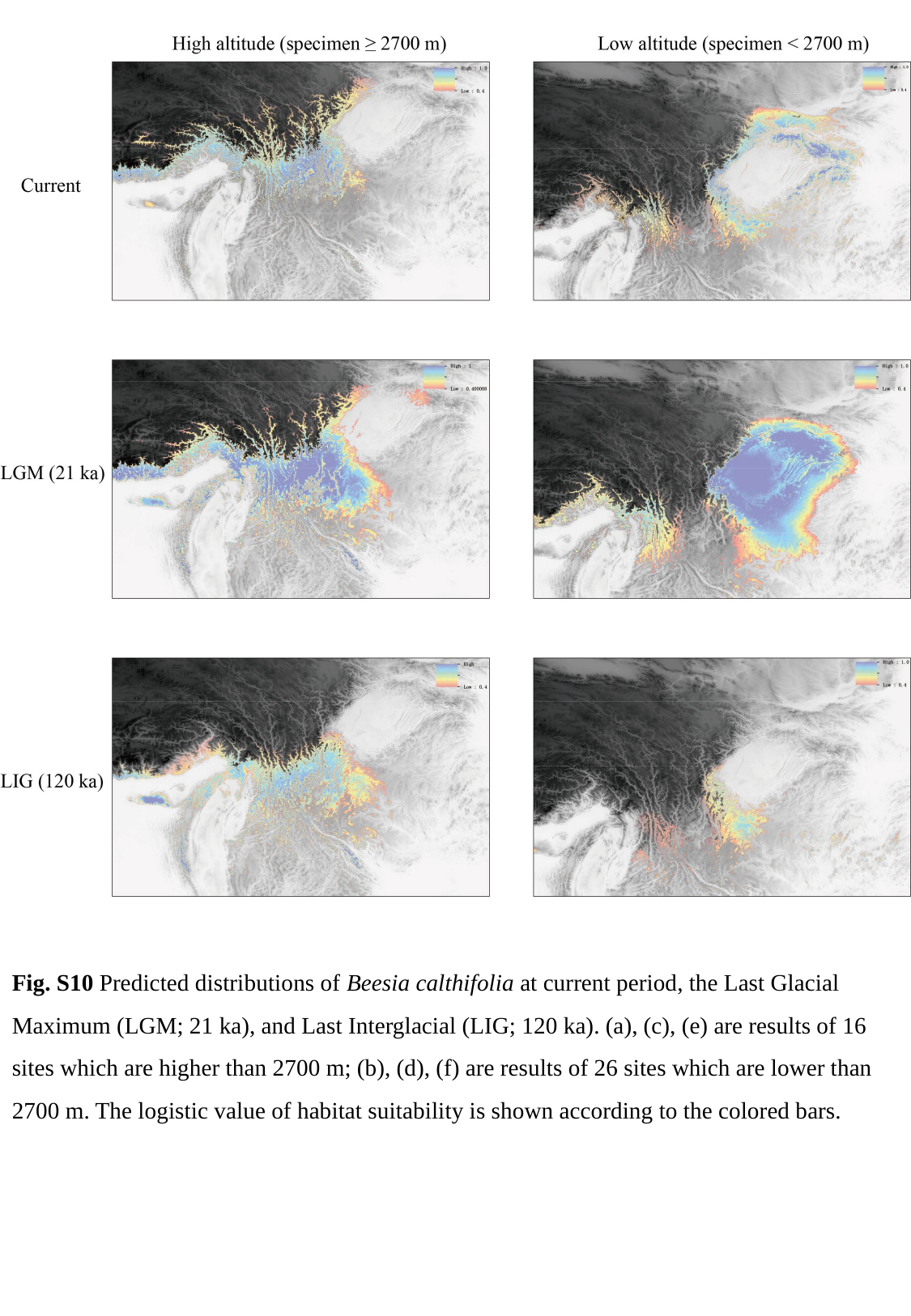

Fig. S10 Predicted distributions of Beesia calthifolia at current period, the Last Glacial Maximum (LGM; 21 ka), and Last Interglacial (LIG; 120 ka). (a), (c), (e) are results of 16 sites which are higher than 2700 m; (b), (d), (f) are results of 26 sites which are lower than 2700 m. The logistic value of habitat suitability is shown according to the colored bars.

## Slide 11
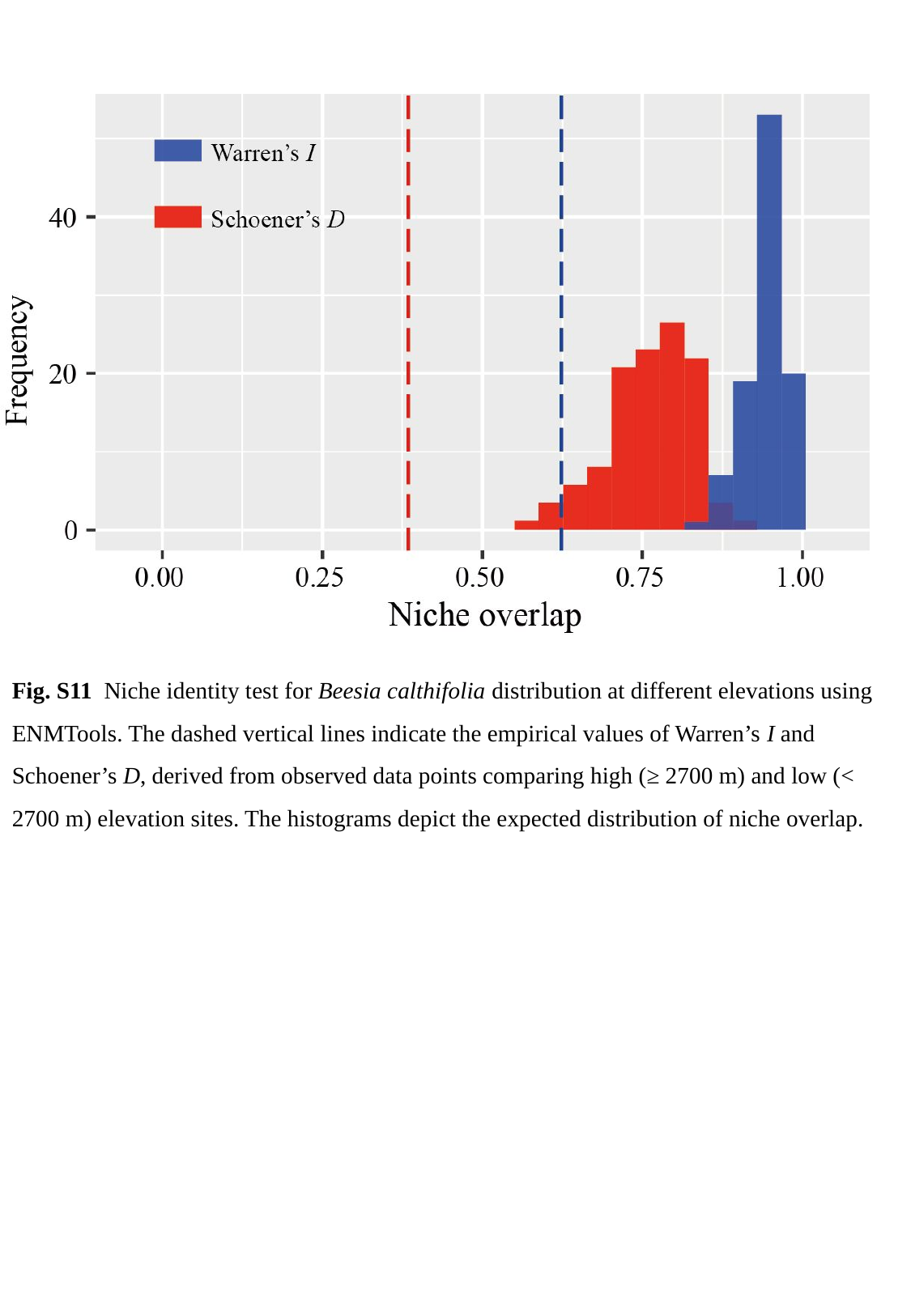

Fig. S11 Niche identity test for Beesia calthifolia distribution at different elevations using ENMTools. The dashed vertical lines indicate the empirical values of Warren’s I and Schoener’s D, derived from observed data points comparing high (≥ 2700 m) and low (< 2700 m) elevation sites. The histograms depict the expected distribution of niche overlap.
