# Supplementary material for: Ancient allopatry and ecological divergence act together to promote plant diversity in mountainous regions: evidence from comparative phylogeography of two genera in the Sino-Himalayan region
Source: BMC Plant Biol. 2023 Nov 17;23:572. doi: 10.1186/s12870-023-04593-1 (PMC10655281; doi:10.1186/s12870-023-04593-1)
Supplement: Supplementary file 2 — Supplementary Material 2 [file 12870_2023_4593_MOESM2_ESM.doc]

**Table S1 Details of sample locations, voucher numbers, and sample sizes for *Beesia*.**

| Species | Population | Locations | n | Geographic coordinates | Altitude (m) | Voucher/collection number |
| --- | --- | --- | --- | --- | --- | --- |
| *B*. *deltophylla* | BD1 | Nyingchi, Medog, Mo Tuo Cun,Tibet | 10 | 29.31°N, 95.36°E | 1908 | ZQP-199, Z. Zhou et al. (KUN) |
| *B*. *deltophylla* | BD2 | Hanmi, Medog, Nyingchi, Tibet | 8 | 29.37°N, 95.11°E | 2250 | YLZB1158, B. Xu & X. H. Xiong (Photo seen) |
| *B*. *deltophylla* | BD3 | 80K, Motuo Hwy, Medog, Nyingchi, Tibet | 10 | 29.7°N, 95.52°E | 2438 | ZQP-227, Z. Zhou et al. (KUN) |
| *B*. *calthifolia* | BC1 | Dujiangyan, Chengdu, Sichuan | 5 | 31.14°N, 103.58°E | 1700 | ZYZ-djy, Y. Z. Zhang (Planted in KIB) |
| *B*. *calthifolia* | BC2 | Yiliang, Zhaotong, Yunnan | 5 | 27.83°N, 104.29°E | 1800 | ZP-135, Z. Zhou & J. C. Peng (KUN) |
| *B*. *calthifolia* | BC3 | Shimen,Changde, Hunan | 11 | 30.11°N, 110.8°E | 2000 | ZDG-shimen, D. G. Zhang (Planted in KIB) |
| *B*. *calthifolia* | BC4 | Dongla Mountain, Baoxing, Sichuan | 8 | 30.43°N, 102.56°E | 2100 | Niu-Baoxing-014, Y. Niu (KUN) |
| *B*. *calthifolia* | BC5 | Emeishan, Leshan, Sichuan | 9 | 29.56°N, 103.34°E | 2156 | ZYZ-emei, Y. Z. Zhang (Photo seen) |
| *B*. *calthifolia* | BC6 | Lushui, Nujiang, Yunnan | 9 | 25.99°N, 98.66°E | 2664 | PSL-002, J. C. Peng & L. Sun (Planted in KIB) |
| *B*. *calthifolia* | BC7 | Mianning, Liangshan, Sichuan | 8 | 28.96°N, 102.16°E | 2899 | ZP-53, Z. Zhou & J. C. Peng (KUN) |
| *B*. *calthifolia* | BC8 | Luding, Garze, Sichuan | 9 | 29.58°N, 102°E | 2903 | ZP-99, Z. Zhou & J. C. Peng (KUN) |
| *B*. *calthifolia* | BC9 | Xizhouzhen, Dali, Yunnan | 2 | 25.87°N, 100°E | 3092 | PSL-001, J. C. Peng & L. Sun (Planted in KIB) |
| *B*. *calthifolia* | BC10 | Kangding, Garze, Sichuan | 10 | 30.17°N, 101.87°E | 3474 | ZP-92, Z. Zhou & J. C. Peng (KUN) |
| *B*. *calthifolia* | BC11 | Muli, Liangshan, Sichuan | 6 | 28.14°N, 101.16°E | 3691 | ZP-22, Z. Zhou & J. C. Peng (KUN) |
| *B*. *calthifolia* | BC12 | Bi Gu Tianchi,Shangri-La, Deqen, Yunnan | 9 | 27.63°N, 99.64°E | 3900 | ZQP-295, Z. Zhou et al. (KUN) |

**Table S2 Details of sample locations, voucher numbers, and sample sizes for *Megacodon*.**

| Species | Population | Locations | n | Geographic coordinates | Altitude (m) | Voucher/collection number |
| --- | --- | --- | --- | --- | --- | --- |
| *M*. *stylophorus* | M1 | Sejila, Linzhi county, Tibet | 22 | 29.57°N, 94.58°E | 3930 | ZQP-257; J. C. Peng et al. (KUN) |
| *M*. *stylophorus* | M2 | Nanyigou, Linzhi county, Tibet | 11 | 29.18°N, 94.22°E | 3800 | Zhouzhuo2017708; Z. Zhou (photo seen) |
| *M*. *stylophorus* | M3 | Lang County, Tibet | 11 | 28.97°N, 93.38°E | 3900 | Zhouzhuo2017709; Z. Zhou (photo seen) |
| *M*. *stylophorus* | M4 | Weixi County, Yunnan | 17 | 28.07°N, 98.76°E | 3875 | Niuyang201716 (photo seen); Y. Niu |
| *M*. *stylophorus* | M5 | Bi-Gu-Tian-Chi, Shangri-La, Yunnan | 15 | 27.63°N, 99.64°E | 3900 | ZQP-294; J. C. Peng et al. (KUN) |
| *M*. *stylophorus* | M6 | Tianbao Mountain, Yunnan | 12 | 27.66°N, 99.92°E | 4000 | YZC-283; H. L. Chen et al. (KUN) |
| *M*. *stylophorus* | M7 | Cangshan, Yunnan | 14 | 25.68°N, 100.09°E | 3808 | ZQP-296; J. C. Peng et al. (KUN) |
| *M*. *stylophorus* | M9 | Bai-Mang-Xue-Shan,Deqin County, Yunnan | 2 | 28.39°N, 98.99°E | 3995 | ZQP-274; J. C. Peng et al. (KUN) |
| *M*. *stylophorus* | M11 | Yagong Mountain, Yunnan | 3 | 28.09°N, 99.52°E | 3920 | CHY021 (photo seen); H. L. Chen |
| *M*. *stylophorus* | M12 | Haba Mountain, Yunnan | 11 | 27.35°N, 100.11°E | 4000 | CP29; H. L. Chen & J. C. Peng (KUN) |
| *M*. *stylophorus* | M13 | Da-Xue-Shan, Yunnan | 12 | 28.59°N, 99.85°E | 4100 | CP50; H. L. Chen & J. C. Peng (KUN) |
| *M*. *stylophorus* | M14 | Longzi County, Tibet | 11 | 28.69°N, 93.27°E | 3690 | ZJW5509; Z. W. Zhang et al. (KUN) |
| *M*. *stylophorus* | M15 | Cuona County, Tibet | 6 | 27.92°N, 91.84°E | 3910 | ZJW5516; Z. W. Zhang et al. (KUN) |
| *M*. *lushuiensis* | M8 | Laowo, Lushui,Yunnan | 14 | 25.85°N, 99.03°E | 1700 | PM001; J. C. Peng & L. Sun (KUN) |
| *M*. *venosus* | M10 | Wuxi, Chongqing | 10 | 31.40°N, 109.76°E | 1109 | PJC001; J. C. Peng & Y. Yang (KUN) |

Table S3 Statistics for RAD-seq of *Megacodon*.

| Ind_ID | Depth of coverage (X) | Total reads | Ind_ID | Depth of coverage (X) | Total reads |
| --- | --- | --- | --- | --- | --- |
| M1_10 | 12.71 | 3654071 | M7_11 | 12.79 | 4012910 |
| M1_11 | 18.96 | 8406549 | M7_1 | 10.43 | 4907403 |
| M1_1 | 18.49 | 7654086 | M7_12 | 15.72 | 5287817 |
| M1_12 | 19.18 | 8642617 | M7_13 | 9.03 | 1349667 |
| M1_13 | 14.54 | 5393326 | M7_14 | 17.45 | 6331761 |
| M1_14 | 21.45 | 11695025 | M7_15 | 17.32 | 6807549 |
| M1_15 | 16.16 | 6097788 | M7_16 | 13.87 | 4096944 |
| M1_16 | 22.14 | 11939610 | M7_3 | 14.81 | 5999569 |
| M1_17 | 21.96 | 11180212 | M7_4 | 17.68 | 7546085 |
| M1_18 | 10.72 | 2456517 | M7_5 | 16.73 | 5947528 |
| M1_19 | 18.45 | 8737206 | M7_6 | 12.34 | 4584387 |
| M1_20 | 15.81 | 6098934 | M7_7 | 18.58 | 6443021 |
| M1_21 | 16.92 | 6594390 | M7_8 | 14.79 | 7400317 |
| M1_2 | 14.87 | 5025845 | M10_10 | 10.93 | 3728325 |
| M1_22 | 13.15 | 3534420 | M10_11 | 12.95 | 4755850 |
| M1_3 | 14.18 | 4320882 | M10_1 | 12.47 | 4956209 |
| M1_4 | 14.53 | 4960642 | M10_2 | 11.68 | 4097650 |
| M1_5 | 15.66 | 5568728 | M10_3 | 8.75 | 5189963 |
| M1_6 | 16.27 | 6411383 | M10_4 | 9.73 | 5663872 |
| M1_7 | 17.17 | 7127018 | M10_5 | 11.62 | 4710650 |
| M1_8 | 14.44 | 4773493 | M10_7 | 10.06 | 3320977 |
| M1_9 | 14.82 | 4963779 | M10_8 | 10.5 | 4105430 |
| M2_10 | 7.66 | 3475551 | M10_9 | 13.34 | 3927869 |
| M2_12 | 13.49 | 6502957 | M8_10 | 8.81 | 8536772 |
| M2_13 | 13.22 | 6371584 | M8_11 | 11.96 | 3578080 |
| M2_14 | 13.88 | 6325219 | M8_1 | 8.11 | 5071914 |
| M2_15 | 12.21 | 6834471 | M8_12 | 7.31 | 5848206 |
| M2_18 | 12.54 | 5484366 | M8_13 | 9.96 | 5243920 |
| M2_3 | 14.16 | 5718049 | M8_14 | 9.58 | 6395763 |
| M2_4 | 14.69 | 6988047 | M8_2 | 10.02 | 5103350 |
| M2_5 | 15.69 | 6753755 | M8_3 | 6.73 | 4822750 |
| M2_6 | 15.22 | 6820915 | M8_4 | 7.27 | 2780713 |
| M2_8 | 12.96 | 5557120 | M8_5 | 7.48 | 11930082 |
| M6_11 | 13.53 | 4051157 | M8_6 | 8.35 | 8591061 |
| M6_1 | 14.12 | 5764924 | M8_7 | 7.54 | 5955022 |
| M6_12 | 13.97 | 4205797 | M8_8 | 9.37 | 5771670 |
| M6_13 | 14.22 | 4832938 | M8_9 | 11.2 | 5579867 |
| M6_14 | 13.54 | 3571845 | M12_10 | 14.46 | 5752531 |
| M6_2 | 17.91 | 5797024 | M12_11 | 12.68 | 4610229 |
| M6_3 | 17.21 | 4753302 | M12_1 | 12.2 | 4886672 |
| M6_5 | 18.94 | 6357984 | M12_12 | 14.32 | 5293611 |
| M6_6 | 17.09 | 4807087 | M12_2 | 11.93 | 3803187 |
| M6_7 | 14.84 | 4145416 | M12_3 | 12.17 | 4089304 |
| M6_8 | 15.33 | 4038891 | M12_4 | 12.72 | 4800227 |
| M6_9 | 13.82 | 3169392 | M12_5 | 14.34 | 5357425 |
| M5_10 | 13.48 | 5759644 | M12_6 | 9.88 | 3395538 |
| M5_11 | 13.94 | 6140214 | M12_8 | 11.79 | 3635400 |
| M5_1 | 11.44 | 4289609 | M12_9 | 13.79 | 4836413 |
| M5_12 | 13.65 | 6213733 | M13_1 | 12.78 | 4027998 |
| M5_13 | 14.58 | 7174740 | M13_2 | 14.64 | 6397421 |
| M5_14 | 17.03 | 9195848 | M13_3 | 15.25 | 6597887 |
| M5_20 | 14.56 | 6853079 | M13_4 | 15.98 | 7942347 |
| M5_2 | 14.02 | 6490490 | M13_5 | 13.44 | 4495636 |
| M5_3 | 13.76 | 5989087 | M13_6 | 13.92 | 4645015 |
| M5_4 | 15.4 | 8431820 | M13_7 | 14.93 | 5978160 |
| M5_5 | 12.79 | 4779936 | M13_8 | 16.7 | 7751415 |
| M5_6 | 14.41 | 6678719 | M13_9 | 14.13 | 5212618 |
| M5_7 | 14.24 | 6276204 | M13_10 | 16.57 | 6788280 |
| M5_8 | 12.39 | 5230291 | M13_11 | 15.16 | 4849629 |
| M5_9 | 12.75 | 5171391 | M13_12 | 17.47 | 6941548 |
| M4_10 | 13.94 | 4787031 | M14_10 | 12.63 | 5490999 |
| M4_11 | 13.06 | 4370189 | M14_1 | 12.97 | 5016762 |
| M4_1 | 15.32 | 6791167 | M14_12 | 13.23 | 4432584 |
| M4_12 | 14.48 | 6057195 | M14_2 | 15.23 | 5461967 |
| M4_13 | 13.83 | 5462308 | M14_3 | 13.16 | 5525344 |
| M4_14 | 11.74 | 4014528 | M14_4 | 16.46 | 6657589 |
| M4_15 | 14.45 | 5656451 | M14_5 | 12.17 | 6865361 |
| M4_16 | 13.75 | 5505130 | M14_6 | 15.8 | 5326011 |
| M4_17 | 13.67 | 5608013 | M14_7 | 17.05 | 5777970 |
| M4_18 | 12.44 | 4031213 | M14_8 | 15.4 | 5534711 |
| M4_19 | 12.93 | 4704433 | M14_9 | 14.76 | 4639590 |
| M4_2 | 13.34 | 4642788 | M15_1 | 16.67 | 9038250 |
| M4_3 | 15.62 | 7444254 | M15_2 | 13.44 | 5056355 |
| M4_4 | 15.45 | 6990453 | M15_3 | 14.31 | 6089715 |
| M4_5 | 15.38 | 6780477 | M15_4 | 14.71 | 6071126 |
| M4_6 | 13.8 | 5747882 | M15_5 | 16.54 | 7694655 |
| M4_9 | 11.17 | 2831452 | M15_6 | 11.33 | 3951952 |
| M7_10 | 16.42 | 6630145 | Total | // | 881394581 |

Table S4 Statistics for RAD-seq of *Beesia*.

| Ind_ID | Depth of coverage (X) | Total_reads | Ind_ID | Depth of coverage (X) | Total_reads |
| --- | --- | --- | --- | --- | --- |
| BC4-1 | 7.88 | 11293369 | BC11-5 | 13.46 | 15826579 |
| BC4-2 | 7.82 | 11034643 | BC11-7 | 12.86 | 7324926 |
| BC4-3 | 7.63 | 10368037 | BC11-8 | 13.2 | 6375421 |
| BC4-4 | 7.52 | 7779410 | BC7-1 | 14.23 | 14240974 |
| BC4-5 | 8.49 | 12501007 | BC7-2 | 12.82 | 8085701 |
| BC4-6 | 7.74 | 9057260 | BC7-3 | 13.72 | 18395921 |
| BC4-7 | 7.29 | 5438423 | BC7-4 | 13.46 | 12562406 |
| BC4-9 | 7.98 | 15253095 | BC7-5 | 14.08 | 12188556 |
| BD1-1 | 7.34 | 7068414 | BC7-6 | 13.51 | 11302983 |
| BD1-3 | 7.38 | 6276577 | BC7-7 | 14.74 | 22917403 |
| BD1-4 | 7.35 | 10528232 | BC7-10 | 13.85 | 16061771 |
| BD1-5 | 7.64 | 8027576 | BC10-1 | 14.15 | 12946128 |
| BD1-6 | 7.49 | 9194084 | BC10-2 | 9.93 | 13375663 |
| BD1-7 | 8.38 | 14197804 | BC10-3 | 8.69 | 8493967 |
| BD1-8 | 9.15 | 13557515 | BC10-4 | 10.19 | 16631003 |
| BD1-9 | 8.91 | 16555784 | BC10-5 | 8.74 | 10534326 |
| BD1-11 | 8.33 | 11818952 | BC10-6 | 8.89 | 10656450 |
| BD1-12 | 7.27 | 10916323 | BC10-7 | 8.82 | 11763565 |
| BC12-1 | 9.35 | 12557304 | BC10-8 | 8.66 | 9914909 |
| BC12-2 | 8.94 | 9088964 | BC10-9 | 7.86 | 6636829 |
| BC12-3 | 10.24 | 15206042 | BC10-10 | 7.84 | 6456574 |
| BC12-4 | 9.21 | 9001103 | BC8-1 | 7.91 | 7919610 |
| BC12-5 | 9.84 | 12628441 | BC8-2 | 8.38 | 7433404 |
| BC12-6 | 9.89 | 13885311 | BC8-3 | 7.62 | 8021406 |
| BC12-7 | 9.08 | 10454726 | BC8-4 | 9.33 | 13188107 |
| BC12-8 | 10.02 | 17997102 | BC8-5 | 8.46 | 10610949 |
| BC12-9 | 8.94 | 8694891 | BC8-6 | 8.78 | 14563308 |
| BD3-1 | 8.36 | 8215314 | BC8-7 | 8.8 | 14569738 |
| BD3-2 | 8.39 | 12690219 | BC8-8 | 9.24 | 8433237 |
| BD3-3 | 8.47 | 6664119 | BC8-9 | 7.94 | 29321250 |
| BD3-4 | 8.27 | 6871719 | BC1-2 | 10.26 | 13741196 |
| BD3-5 | 8.30 | 6831420 | BC1-3 | 8.55 | 9998465 |
| BD3-6 | 8.28 | 9610019 | BC1-4 | 9.98 | 11904170 |
| BD3-7 | 8.61 | 8653686 | BC1-5 | 10.57 | 10443765 |
| BD3-8 | 9.22 | 12629984 | BC1-9 | 8.42 | 13716324 |
| BD3-9 | 7.86 | 6540633 | BC2-1 | 9 | 16966296 |
| BD3-10 | 8.39 | 7178974 | BC2-3 | 7.75 | 26789368 |
| BC5-1 | 8.79 | 10893702 | BC2-4 | 7.12 | 30195943 |
| BC5-2 | 13.47 | 10417971 | BC2-6 | 6.77 | 7328754 |
| BC5-4 | 12.83 | 8161286 | BC2-7 | 6.52 | 5530705 |
| BC5-5 | 12.22 | 6021836 | BC9-1 | 6.04 | 5671119 |
| BC5-6 | 12.95 | 7474116 | BC9-2 | 6.17 | 5067610 |
| BC5-7 | 12.96 | 7573272 | BC6-4 | 7.08 | 9688102 |
| BC5-8 | 13.07 | 8017762 | BC6-6 | 6.86 | 6985633 |
| BC5-9 | 13.85 | 10969739 | BC6-8 | 6.12 | 5474131 |
| BC5-10 | 14.35 | 18047660 | BC6-9 | 13.64 | 14163635 |
| BC3-1 | 13.29 | 11104659 | BC6-10 | 14.55 | 15807104 |
| BC3-2 | 12.80 | 7574050 | BC6-12 | 15.49 | 21392243 |
| BC3-3 | 13.67 | 9447158 | BC6-13 | 13.86 | 11691746 |
| BC3-4 | 13.72 | 7256047 | BC6-14 | 14.1 | 11328949 |
| BC3-5 | 12.33 | 8738364 | BC6-15 | 14.65 | 17741173 |
| BC3-6 | 12.79 | 7399541 | BD2-3 | 12.61 | 4426602 |
| BC3-7 | 12.52 | 6753953 | BD2-4 | 12.56 | 5918415 |
| BC3-8 | 12.68 | 8221409 | BD2-5 | 12.62 | 5340887 |
| BC3-9 | 12.06 | 8819128 | BD2-6 | 12.65 | 7916074 |
| BC3-10 | 12.87 | 9561317 | BD2-7 | 12.95 | 7079064 |
| BC3-11 | 12.64 | 6753741 | BD2-8 | 13.4 | 10922408 |
| BC11-2 | 12.64 | 5117108 | BD2-9 | 12.64 | 4588894 |
| BC11-3 | 13.13 | 7055431 | BD2-10 | 12.88 | 6693853 |
| BC11-4 | 13.84 | 10419764 | Total | // | 1279331182 |

Table S5 Summary of genetic diversity statistics within populations of *Beesia* based on dataset B119.

| Group ID | Pop ID | n | Private (%) | *H*_obs_ | π | *F*_IS_ | Polymorphic (%) |
| --- | --- | --- | --- | --- | --- | --- | --- |
| BD_EH | BD1 | 10 | 1.65 | 0.021 | 0.020 | 0.005 | 0.04 |
|  | BD2 | 8 | 0.90 | 0.023 | 0.026 | 0.010 | 0.06 |
|  | BD3 | 10 | 1.05 | 0.019 | 0.013 | -0.012 | 0.03 |
| BC_QCS | BC1 | 5 | 14.11 | 0.022 | 0.017 | -0.006 | 0.04 |
| BC_SC | BC2 | 5 | 1.35 | 0.021 | 0.023 | 0.005 | 0.05 |
|  | BC3 | 11 | 2.70 | 0.013 | 0.020 | 0.019 | 0.05 |
|  | BC4 | 8 | 4.65 | 0.019 | 0.018 | -0.002 | 0.04 |
|  | BC5 | 9 | 2.40 | 0.023 | 0.020 | -0.008 | 0.03 |
| BC_LS | BC6 | 9 | 6.91 | 0.017 | 0.017 | 0.002 | 0.06 |
| BC_HM | BC7 | 8 | 0.75 | 0.015 | 0.026 | 0.028 | 0.06 |
|  | BC8 | 9 | 0.30 | 0.013 | 0.019 | 0.013 | 0.04 |
|  | BC9 | 2 | 0.75 | 0.062 | 0.051 | -0.017 | 0.06 |
|  | BC10 | 10 | 1.05 | 0.017 | 0.019 | 0.009 | 0.06 |
|  | BC11 | 6 | 2.85 | 0.014 | 0.020 | 0.011 | 0.04 |
|  | BC12 | 9 | 1.80 | 0.016 | 0.017 | 0.002 | 0.04 |

Table S6 Summary of genetic diversity statistics within species of *Megacodon* based on dataset M155.

| Species ID | n | Private (%) | *H*_obs_ | π | *F*_IS_ | Polymorphic (%) |
| --- | --- | --- | --- | --- | --- | --- |
| *M. stylophorus* | 131 | 46.8 | 0.025 | 0.101 | 0.25898 | 0.36 |
| *M. venosus* | 10 | 17.1 | 0.069 | 0.051 | -0.03745 | 0.13 |
| *M. lushuiensis* | 14 | 11.4 | 0.084 | 0.080 | -0.00907 | 0.22 |

**Table S7 Summary of genetic diversity statistics within populations of *Megacodon stylophorus* based on dataset M131.**

| Group ID | Pop ID | n | Private (%) | *H*_obs_ | π | *F*_IS_ | Polymorphic (%) |
| --- | --- | --- | --- | --- | --- | --- | --- |
| MS_EH1 | M1 | 21 | 0.3 | 0.023 | 0.020 | -0.004 | 0.048 |
|  | M2 | 11 | 2.1 | 0.040 | 0.043 | 0.008 | 0.082 |
|  | M14 | 11 | 3.5 | 0.028 | 0.024 | -0.007 | 0.093 |
| MS_EH2 | M15 | 6 | 13.8 | 0.041 | 0.037 | -0.005 | 0.108 |
| MS_HM1 | M4 | 15 | 7.9 | 0.043 | 0.046 | 0.010 | 0.152 |
|  | M7 | 13 | 6.8 | 0.032 | 0.029 | -0.004 | 0.104 |
| MS_HM2 | M6 | 12 | 1.1 | 0.042 | 0.043 | 0.006 | 0.069 |
|  | M5 | 15 | 6.3 | 0.040 | 0.044 | 0.015 | 0.066 |
|  | M12 | 11 | 1.9 | 0.036 | 0.038 | 0.006 | 0.046 |
|  | M13 | 12 | 0.5 | 0.035 | 0.033 | -0.002 | 0.055 |

Table S8 Results of standard and partial Mantel tests on genetic, geographic, elevational, and climatic distance matrix of populations from different groups of *Beesia*

| Groups included | Matrix A | Matrix B | Adjustment | Mantel's r | *P*-value |
| --- | --- | --- | --- | --- | --- |
| *Beesia* | Genetic | Geographic | - | 0.403 | 0.004 |
|  | Genetic | Climatic | - | 0.108 | 0.236 |
|  | Genetic | Elevational | - | 0.238 | 0.037 |
|  | Genetic | Geographic | Partial: Climatic | 0.415 | 0.002 |
|  | Genetic | Geographic | Partial: Elevational | 0.437 | 0.002 |
|  | Genetic | Climatic | Partial: Geographic | -0.154 | 0.860 |
|  | Genetic | Climatic | Partial: Elevational | 0.038 | 0.374 |
|  | Genetic | Elevational | Partial: Geographic | 0.298 | 0.016 |
|  | Genetic | Elevational | Partial: Climatic | 0.216 | 0.057 |
| BC2-BC12 | Genetic | Geographic | - | 0.170 | 0.186 |
|  | Genetic | Climatic | - | 0.393 | 0.006 |
|  | Genetic | Elevational | - | 0.547 | 0.001 |
|  | Genetic | Geographic | Partial: Climatic | -0.219 | 0.930 |
|  | Genetic | Geographic | Partial: Elevational | 0.109 | 0.294 |
|  | Genetic | Climatic | Partial: Geographic | 0.413 | 0.005 |
|  | Genetic | Climatic | Partial: Elevational | 0.213 | 0.121 |
|  | Genetic | Elevational | Partial: Geographic | 0.536 | 0.001 |
|  | Genetic | Elevational | Partial: Climatic | 0.457 | 0.003 |
| *M*. *stylophorus* | Genetic | Geographic | - | 0.852 | 0.001 |
|  | Genetic | Climatic | - | 0.145 | 0.179 |
|  | Genetic | Elevational | - | 0.082 | 0.300 |
|  | Genetic | Geographic | Partial: Climatic | 0.849 | 0.000 |
|  | Genetic | Geographic | Partial: Elevational | 0.851 | 0.000 |
|  | Genetic | Climatic | Partial: Geographic | 0.064 | 0.321 |
|  | Genetic | Climatic | Partial: Elevational | 0.175 | 0.138 |
|  | Genetic | Elevational | Partial: Geographic | 0.129 | 0.228 |
|  | Genetic | Elevational | Partial: Climatic | 0.036 | 0.400 |

Table S9 Outgroups and sister groups used for RAxML and Beast analysis based on whole chloroplast genome (WCG).

| Species | GenBank Accession |
| --- | --- |
| *Eranthis stellata* | NC_041536 |
| *Actaea asiatica* | MK569469 |
| *Actaea dahurica* | MK253463 |
| *Actaea vaginata* | MK253451 |
| *Anemonopsis macrophylla* | NC_041527 |
| *Beesia calthifolia* | MK253467 |

Table S10 Bioclimatic variables and results of t-test for BC_HM and BC_SC groups of *Beesia*.

|  | | Group | BIO1 | BIO2 | BIO3 | BIO4 | BIO5 | BIO6 | BIO7 | BIO8 | BIO9 | BIO10 | BIO11 | BIO12 | BIO13 | BIO14 | BIO15 | BIO16 | BIO17 | BIO18 | BIO19 |
| --- | --- | --- | --- | --- | --- | --- | --- | --- | --- | --- | --- | --- | --- | --- | --- | --- | --- | --- | --- | --- | --- |
| BD1 | BD_EH | | 144 | 118 | 44 | 5225 | 261 | -5 | 266 | 205 | 81 | 205 | 71 | 1525 | 325 | 6 | 92 | 909 | 28 | 909 | 39 |
| BD2 | BD_EH | | 110 | 121 | 45 | 5282 | 231 | -39 | 269 | 173 | 47 | 173 | 38 | 1001 | 205 | 3 | 90 | 579 | 18 | 579 | 21 |
| BD3 | BD_EH | | 108 | 125 | 44 | 5510 | 233 | -47 | 279 | 173 | 42 | 173 | 33 | 963 | 202 | 4 | 91 | 566 | 18 | 566 | 21 |
| BC1 | BC_QCS | | 108 | 94 | 33 | 6265 | 239 | -38 | 277 | 180 | 22 | 185 | 22 | 998 | 201 | 7 | 81 | 532 | 27 | 528 | 27 |
| BC2 | BC_SC | | 116 | 87 | 33 | 6032 | 243 | -19 | 262 | 189 | 32 | 189 | 32 | 917 | 175 | 12 | 78 | 492 | 38 | 492 | 38 |
| BC3 | BC_SC | | 97 | 69 | 24 | 7379 | 236 | -45 | 281 | 170 | -1 | 190 | -1 | 1509 | 220 | 33 | 53 | 622 | 116 | 611 | 116 |
| BC4 | BC_SC | | 101 | 87 | 32 | 6082 | 226 | -36 | 262 | 175 | 17 | 175 | 17 | 877 | 152 | 6 | 77 | 437 | 24 | 437 | 24 |
| BC5 | BC_SC | | 109 | 75 | 29 | 6114 | 232 | -22 | 254 | 183 | 26 | 185 | 26 | 1257 | 283 | 11 | 87 | 706 | 40 | 702 | 40 |
| BC6 | BC_LS | | 122 | 110 | 48 | 4340 | 208 | -20 | 227 | 172 | 62 | 172 | 62 | 1371 | 287 | 12 | 83 | 770 | 64 | 770 | 64 |
| BC7 | BC_HM | | 80 | 115 | 43 | 5230 | 194 | -73 | 266 | 143 | 8 | 143 | 8 | 1083 | 218 | 5 | 89 | 613 | 21 | 613 | 21 |
| BC8 | BC_HM | | 55 | 115 | 41 | 5555 | 176 | -101 | 277 | 123 | -21 | 123 | -21 | 994 | 196 | 5 | 88 | 554 | 21 | 554 | 21 |
| BC9 | BC_HM | | 88 | 110 | 48 | 4243 | 177 | -48 | 225 | 138 | 30 | 138 | 30 | 1084 | 242 | 8 | 94 | 650 | 35 | 650 | 35 |
| BC10 | BC_HM | | 27 | 125 | 42 | 5824 | 153 | -142 | 295 | 97 | -53 | 97 | -53 | 935 | 186 | 5 | 90 | 536 | 20 | 536 | 20 |
| BC11 | BC_HM | | 59 | 133 | 48 | 5077 | 169 | -107 | 276 | 119 | -10 | 119 | -10 | 915 | 228 | 3 | 102 | 584 | 12 | 584 | 12 |
| BC12 | BC_HM | | 43 | 113 | 45 | 5003 | 147 | -102 | 249 | 103 | -24 | 103 | -24 | 824 | 200 | 6 | 94 | 503 | 25 | 503 | 25 |
| BC_HM vs. BC_SC | | | ** | ** | ** | * | ** | ** | // | ** | // | ** | // | // | // | // | * | // | // | // | // |

Note: *: P-value <0.05, **: P-value <0.01, //: P-value >0.05.

**Table S11** Data size of resequencing and sequencing depth, length, and GC content of whole chloroplast genome (WCG) of *Beesia*.

| ID | Clean data (G) | Coverage (X) | WCG length (bp) | GC content | GenBank Accession |
| --- | --- | --- | --- | --- | --- |
| BC1-2 | 5.6 | 31.4 | 157,672 | 38.30% | OQ145146 |
| BC1-3 | 5.8 | 48.9 | 157,480 | 38.40% | OQ145147 |
| BC1-4 | 5.2 | 35.2 | 157,380 | 38.40% | OQ145148 |
| BC2-1 | 5.2 | 62.1 | 158,085 | 38.20% | OQ145152 |
| BC4-4 | 5.6 | 106.2 | 156,961 | 38.30% | OQ145153 |
| BC5-3 | 5.6 | 21.4 | 158,114 | 38.20% | OQ145154 |
| BC6-1 | 5.4 | 52.4 | 158,274 | 38.20% | OQ145155 |
| BC7-3 | 5.8 | 63.5 | 158,099 | 38.20% | OQ145156 |
| BC8-1 | 6 | 58.9 | 158,115 | 38.20% | OQ145157 |
| BC9-1 | 6.4 | 53.1 | 158,214 | 38.20% | OQ145158 |
| BC10-1 | 6 | 85.1 | 158,132 | 38.20% | OQ145149 |
| BC11-1 | 5.2 | 81.8 | 158,170 | 38.20% | OQ145150 |
| BC12-1 | 6.8 | 224.1 | 158,107 | 38.20% | OQ145151 |
| BD1-2 | 6.8 | 72.1 | 157,506 | 38.40% | OQ145159 |
| BD2-1 | 5.4 | 96.9 | 157,557 | 38.30% | OQ145160 |
| BD3-1 | 5.2 | 52.7 | 157,397 | 38.40% | OQ145161 |

**Table S12** Estimates of historical gene flow (*N*_m_) among five groups of *Beesia* based on B119 dataset.

|  |  | M | | | | | *N*_m_ | | | | |
| --- | --- | --- | --- | --- | --- | --- | --- | --- | --- | --- | --- |
| Groups ID | Θ | BD_EH → | BC_QCS → | BC_SC → | BC_LS → | BC_HM → | BD_EH → | BC_QCS → | BC_SC → | BC_LS → | BC_HM → |
| BD_EH | 0.00003 | - | 24.6 | 66.6 | 49.9 | 232.5 | - | 0.0002 | 0.0005 | 0.0004 | 0.0017 |
| BC_QCS | 0.00121 | 483.6 | - | 457 | 165.3 | 660.1 | 0.1463 | - | 0.1382 | 0.0500 | 0.1997 |
| BC_SC | 0.00003 | 87.6 | 66.8 | - | 70.3 | 100.3 | 0.0007 | 0.0005 | - | 0.0005 | 0.0008 |
| BC_LS | 0.0591 | 116.7 | 84.8 | 711.5 | - | 715.3 | 1.7 | 1.3 | 10.5 | - | 10.6 |
| BC_HM | 0.00013 | 86.4 | 42.4 | 106.9 | 49.1 | - | 0.0028 | 0.0014 | 0.0035 | 0.0016 | - |

Table S13 Estimates of historical gene flow (*N*_m_) among three species of *Megacodon* based on M155 dataset.

|  |  | M | | | *N*_m_ | | |
| --- | --- | --- | --- | --- | --- | --- | --- |
| Species ID | Θ | *M. lushuiensis*→ | *M. venosus*→ | *M. stylophorus*→ | *M. lushuiensis*→ | *M. venosus*→ | *M. stylophorus*→ |
| *M. lushuiensis* | 0.0016 | - | 35.9 | 62 | - | 0.015 | 0.022 |
| *M. venosus* | 0.0014 | 37.1 | - | 30.8 | 0.013 | - | 0.013 |
| *M. stylophorus* | 0.0018 | 14.3 | 21.4 | - | 0.007 | 0.010 | - |

**Table S14** Estimates of historical gene flow (*N*_m_) among four groups of *M*. *stylophorus* based on M131 dataset.

|  |  | M | | | | *N*_m_ | | | |
| --- | --- | --- | --- | --- | --- | --- | --- | --- | --- |
| Groups ID | Θ | MS_HM1→ | MS_HM2→ | MS_EH1→ | MS_EH2→ | MS_HM1→ | MS_HM2→ | MS_EH1→ | MS_EH2→ |
| MS_HM1 | 0.002 | - | 118.9 | 36.6 | 23 | - | 0.05 | 0.02 | 0.01 |
| MS_HM2 | 0.001 | 204.3 | - | 99 | 56.2 | 0.25 | - | 0.12 | 0.07 |
| MS_EH1 | 0.001 | 56 | 65.7 | - | 40.9 | 0.02 | 0.02 | - | 0.02 |
| MS_EH2 | 0.052 | 590.1 | 456.8 | 591.2 | - | 7.71 | 5.97 | 7.73 | - |

**Table S15** Mean value (mean), standard error (SE), coefficient of variation (CV) of leaf traits evaluated in specimens of *Beesia calthifolia*.

| Leaf traits | 79 individuals (≥ 2700 m) | | 179 individuals (＜ 2700 m) | | All individuals | |
| --- | --- | --- | --- | --- | --- | --- |
|  | Mean ± SE | CV (%) | Mean ± SE | CV (%) | Mean ± SE | CV (%) |
| Leaf width (cm) | 10.7972 ± 0.3125 | 0.2572 | 10.1091 ± 0.2159 | 0.2857 | 10.3198 ± 0.17849 | 0.2778 |
| Leaf length (cm) | 6.7155 ± 0.1869 | 0.2474 | 7.6273 ± 0.1354 | 0.2376 | 7.3481 ± 0.1129 | 0.2468 |
| Leaf area (cm^2^) | 77.4693 ± 4.5125 | 0.5177 | 76.8750 ± 2.9671 | 0.5164 | 77.0570 ± 2.4745 | 0.5158 |
| Number of teeth | 92.3798 ± 2.844 | 0.2737 | 84.4916 ± 2.0475 | 0.3242 | 86.9070 ± 1.6786 | 0.3102 |
| Leaf perimeter (cm) | 35.8536 ± 1.0715 | 0.2656 | 36.4819 ± 0.7033 | 0.2580 | 36.2895 ± 0.5872 | 0.2599 |
| Petiole (cm) | 16.5620 ± 0.4348 | 0.2333 | 16.7990 ± 0.3406 | 0.2712 | 16.7264 ± 0.2708 | 0.2601 |
| Leaf shape (leaf length/leaf width) | 0.6263 ± 0.0079 | 0.1117 | 0.7818 ± 0.0126 | 0.2163 | 0.7342 ± 0.0101 | 0.2215 |
| Tooth density (number of teeth/perimeter) | 2.6646 ± 0.0849 | 0.2834 | 2.3610 ± 0.0491 | 0.2780 | 2.4540 ± 0.0436 | 0.2856 |

| **Table S16** Model and parameters of ancestral range estimation of extant *Beesia* lineages. | | | | | | | |
| --- | --- | --- | --- | --- | --- | --- | --- |
| Model | LnL | nParams | d | e | j | AIC | w |
| DEC | -19.5995 | 2 | 0.053642 | 0.129122 | 0 | 43.20 | 0.00023 |
| DEC+J | -11.467 | 3 | 1.00E-12 | 1.00E-12 | 0.200 | 28.93 | 0.28493 |
| DIVALIKE | -18.1984 | 2 | 0.038266 | 0.053435 | 0 | 40.40 | 0.00092 |
| DIVALIKE+J | -10.7532 | 3 | 1.00E-12 | 1.00E-12 | 0.165 | 27.51 | 0.58171 |
| BAYAREALIKE | -21.291 | 2 | 0.068337 | 0.2272 | 0 | 46.58 | 0.00004 |
| BAYAREALIKE+J | -12.2351 | 3 | 1.00E-07 | 1.00E-07 | 0.200 | 30.47 | 0.13217 |

lnL: log-likelihood; d: rate of range expansion; e: rate of range reduction through extirpation in an area; AIC: Akaike information Criterion; w: Akaike weights.

| **Table S17** Model and parameters of ancestral range estimation of extant *Megacodon* lineages. | | | | | | | |
| --- | --- | --- | --- | --- | --- | --- | --- |
| Model | LnL | nParams | d | e | j | AIC | w |
| DEC | -9.67907 | 2 | 0.047712 | 0.043101 | 0 | 23.36 | 0.01249 |
| DEC+J | -5.96292 | 3 | 1.00E-12 | 1.00E-12 | 0.193 | 17.93 | 0.18894 |
| DIVALIKE | -6.55776 | 2 | 0.040488 | 1.00E-12 | 0 | 17.12 | 0.28333 |
| DIVALIKE+J | -5.16464 | 3 | 1.00E-12 | 1.00E-12 | 0.152 | 16.33 | 0.41977 |
| BAYAREALIKE | -11.8301 | 2 | 0.091173 | 0.234545 | 0 | 27.66 | 0.00145 |
| BAYAREALIKE+J | -6.6609 | 3 | 1.00E-07 | 1.00E-07 | 0.200 | 19.32 | 0.09401 |
|  | | | | | | | |
|  |  |  |  |  |  |  |  |

Table S18 The correlation analysis of bioclimatic variables based on 16 sites from high-elevation specimen (≥ 2700 m) of *Beesia calthifolia*

|  | BIO1 | BIO2 | BIO3 | BIO4 | BIO5 | BIO6 | BIO7 | BIO8 | BIO9 | BIO10 | BIO11 | BIO12 | BIO13 | BIO14 | BIO15 | BIO16 | BIO17 | BIO18 | BIO19 |
| --- | --- | --- | --- | --- | --- | --- | --- | --- | --- | --- | --- | --- | --- | --- | --- | --- | --- | --- | --- |
| BIO1 | 1.00 | -0.26 | -0.07 | -0.11 | 0.93 | 0.97 | -0.32 | 0.98 | 0.98 | 0.98 | 0.97 | 0.74 | 0.64 | 0.13 | -0.02 | 0.69 | 0.28 | 0.69 | 0.54 |
| BIO2 | -0.26 | 1.00 | 0.75 | -0.32 | -0.28 | -0.42 | 0.34 | -0.33 | -0.14 | -0.35 | -0.18 | 0.04 | 0.25 | -0.71 | 0.70 | 0.25 | -0.61 | 0.25 | -0.46 |
| BIO3 | -0.07 | 0.75 | 1.00 | -0.86 | -0.31 | -0.09 | -0.36 | -0.25 | 0.14 | -0.26 | 0.13 | 0.10 | 0.49 | -0.26 | 0.85 | 0.38 | -0.09 | 0.38 | -0.02 |
| BIO4 | -0.11 | -0.32 | -0.86 | 1.00 | 0.23 | -0.19 | 0.77 | 0.09 | -0.32 | 0.11 | -0.34 | -0.14 | -0.53 | -0.16 | -0.69 | -0.38 | -0.33 | -0.38 | -0.31 |
| BIO5 | 0.93 | -0.28 | -0.31 | 0.23 | 1.00 | 0.86 | 0.02 | 0.98 | 0.85 | 0.98 | 0.83 | 0.70 | 0.46 | -0.01 | -0.23 | 0.58 | 0.09 | 0.57 | 0.38 |
| BIO6 | 0.97 | -0.42 | -0.09 | -0.19 | 0.86 | 1.00 | -0.50 | 0.93 | 0.96 | 0.93 | 0.97 | 0.65 | 0.59 | 0.30 | -0.08 | 0.60 | 0.45 | 0.60 | 0.64 |
| BIO7 | -0.32 | 0.34 | -0.36 | 0.77 | 0.02 | -0.50 | 1.00 | -0.16 | -0.44 | -0.16 | -0.48 | -0.09 | -0.36 | -0.60 | -0.23 | -0.20 | -0.72 | -0.20 | -0.61 |
| BIO8 | 0.98 | -0.33 | -0.25 | 0.09 | 0.98 | 0.93 | -0.16 | 1.00 | 0.91 | 1.00 | 0.90 | 0.71 | 0.53 | 0.09 | -0.17 | 0.61 | 0.21 | 0.60 | 0.47 |
| BIO9 | 0.98 | -0.14 | 0.14 | -0.32 | 0.85 | 0.96 | -0.44 | 0.91 | 1.00 | 0.91 | 1.00 | 0.74 | 0.73 | 0.11 | 0.14 | 0.75 | 0.31 | 0.75 | 0.57 |
| BIO10 | 0.98 | -0.35 | -0.26 | 0.11 | 0.98 | 0.93 | -0.16 | 1.00 | 0.91 | 1.00 | 0.90 | 0.71 | 0.52 | 0.10 | -0.18 | 0.60 | 0.22 | 0.60 | 0.47 |
| BIO11 | 0.97 | -0.18 | 0.13 | -0.34 | 0.83 | 0.97 | -0.48 | 0.90 | 1.00 | 0.90 | 1.00 | 0.73 | 0.73 | 0.16 | 0.14 | 0.73 | 0.34 | 0.73 | 0.58 |
| BIO12 | 0.74 | 0.04 | 0.10 | -0.14 | 0.70 | 0.65 | -0.09 | 0.71 | 0.74 | 0.71 | 0.73 | 1.00 | 0.84 | 0.11 | 0.07 | 0.95 | 0.23 | 0.94 | 0.56 |
| BIO13 | 0.64 | 0.25 | 0.49 | -0.53 | 0.46 | 0.59 | -0.36 | 0.53 | 0.73 | 0.52 | 0.73 | 0.84 | 1.00 | 0.06 | 0.54 | 0.96 | 0.20 | 0.96 | 0.48 |
| BIO14 | 0.13 | -0.71 | -0.26 | -0.16 | -0.01 | 0.30 | -0.60 | 0.09 | 0.11 | 0.10 | 0.16 | 0.11 | 0.06 | 1.00 | -0.39 | 0.02 | 0.92 | 0.01 | 0.76 |
| BIO15 | -0.02 | 0.70 | 0.85 | -0.69 | -0.23 | -0.08 | -0.23 | -0.17 | 0.14 | -0.18 | 0.14 | 0.07 | 0.54 | -0.39 | 1.00 | 0.39 | -0.32 | 0.39 | -0.24 |
| BIO16 | 0.69 | 0.25 | 0.38 | -0.38 | 0.58 | 0.60 | -0.20 | 0.61 | 0.75 | 0.60 | 0.73 | 0.95 | 0.96 | 0.02 | 0.39 | 1.00 | 0.16 | 1.00 | 0.49 |
| BIO17 | 0.28 | -0.61 | -0.09 | -0.33 | 0.09 | 0.45 | -0.72 | 0.21 | 0.31 | 0.22 | 0.34 | 0.23 | 0.20 | 0.92 | -0.32 | 0.16 | 1.00 | 0.16 | 0.91 |
| BIO18 | 0.69 | 0.25 | 0.38 | -0.38 | 0.57 | 0.60 | -0.20 | 0.60 | 0.75 | 0.60 | 0.73 | 0.94 | 0.96 | 0.01 | 0.39 | 1.00 | 0.16 | 1.00 | 0.49 |
| BIO19 | 0.54 | -0.46 | -0.02 | -0.31 | 0.38 | 0.64 | -0.61 | 0.47 | 0.57 | 0.47 | 0.58 | 0.56 | 0.48 | 0.76 | -0.24 | 0.49 | 0.91 | 0.49 | 1.00 |

Table S19 The correlation analysis of bioclimatic variables based on 26 sites from low-elevation specimen (< 2700 m) of *Beesia calthifolia*

|  | BIO1 | BIO2 | BIO3 | BIO4 | BIO5 | BIO6 | BIO7 | BIO8 | BIO9 | BIO10 | BIO11 | BIO12 | BIO13 | BIO14 | BIO15 | BIO16 | BIO17 | BIO18 | BIO19 |
| --- | --- | --- | --- | --- | --- | --- | --- | --- | --- | --- | --- | --- | --- | --- | --- | --- | --- | --- | --- |
| BIO1 | 1.00 | -0.16 | -0.21 | 0.23 | 0.85 | 0.91 | 0.16 | 0.91 | 0.86 | 0.88 | 0.86 | 0.05 | -0.01 | 0.12 | -0.17 | 0.01 | 0.10 | -0.01 | 0.10 |
| BIO2 | -0.16 | 1.00 | 0.85 | -0.47 | -0.27 | -0.37 | 0.03 | -0.24 | 0.08 | -0.35 | 0.08 | -0.65 | -0.29 | -0.71 | 0.75 | -0.38 | -0.64 | -0.28 | -0.64 |
| BIO3 | -0.21 | 0.85 | 1.00 | -0.84 | -0.56 | -0.21 | -0.49 | -0.46 | 0.23 | -0.57 | 0.23 | -0.24 | 0.18 | -0.43 | 0.67 | 0.10 | -0.33 | 0.22 | -0.33 |
| BIO4 | 0.23 | -0.47 | -0.84 | 1.00 | 0.69 | 0.01 | 0.86 | 0.56 | -0.30 | 0.66 | -0.30 | -0.16 | -0.53 | 0.10 | -0.45 | -0.47 | 0.01 | -0.60 | 0.01 |
| BIO5 | 0.85 | -0.27 | -0.56 | 0.69 | 1.00 | 0.63 | 0.64 | 0.97 | 0.46 | 0.99 | 0.46 | -0.18 | -0.39 | 0.02 | -0.28 | -0.35 | -0.05 | -0.43 | -0.05 |
| BIO6 | 0.91 | -0.37 | -0.21 | 0.01 | 0.63 | 1.00 | -0.20 | 0.73 | 0.89 | 0.71 | 0.89 | 0.35 | 0.31 | 0.36 | -0.28 | 0.33 | 0.34 | 0.31 | 0.34 |
| BIO7 | 0.16 | 0.03 | -0.49 | 0.86 | 0.64 | -0.20 | 1.00 | 0.50 | -0.30 | 0.54 | -0.30 | -0.57 | -0.79 | -0.33 | -0.07 | -0.77 | -0.40 | -0.85 | -0.40 |
| BIO8 | 0.91 | -0.24 | -0.46 | 0.56 | 0.97 | 0.73 | 0.50 | 1.00 | 0.60 | 0.98 | 0.60 | -0.12 | -0.25 | -0.02 | -0.20 | -0.21 | -0.07 | -0.28 | -0.07 |
| BIO9 | 0.86 | 0.08 | 0.23 | -0.30 | 0.46 | 0.89 | -0.30 | 0.60 | 1.00 | 0.52 | 1.00 | 0.15 | 0.30 | 0.08 | 0.06 | 0.28 | 0.11 | 0.32 | 0.11 |
| BIO10 | 0.88 | -0.35 | -0.57 | 0.66 | 0.99 | 0.71 | 0.54 | 0.98 | 0.52 | 1.00 | 0.52 | -0.03 | -0.25 | 0.13 | -0.35 | -0.21 | 0.07 | -0.29 | 0.07 |
| BIO11 | 0.86 | 0.08 | 0.23 | -0.30 | 0.46 | 0.89 | -0.30 | 0.60 | 1.00 | 0.52 | 1.00 | 0.15 | 0.30 | 0.08 | 0.06 | 0.28 | 0.11 | 0.32 | 0.11 |
| BIO12 | 0.05 | -0.65 | -0.24 | -0.16 | -0.18 | 0.35 | -0.57 | -0.12 | 0.15 | -0.03 | 0.15 | 1.00 | 0.83 | 0.85 | -0.67 | 0.88 | 0.90 | 0.83 | 0.90 |
| BIO13 | -0.01 | -0.29 | 0.18 | -0.53 | -0.39 | 0.31 | -0.79 | -0.25 | 0.30 | -0.25 | 0.30 | 0.83 | 1.00 | 0.51 | -0.17 | 0.98 | 0.59 | 0.98 | 0.59 |
| BIO14 | 0.12 | -0.71 | -0.43 | 0.10 | 0.02 | 0.36 | -0.33 | -0.02 | 0.08 | 0.13 | 0.08 | 0.85 | 0.51 | 1.00 | -0.86 | 0.56 | 0.99 | 0.51 | 0.99 |
| BIO15 | -0.17 | 0.75 | 0.67 | -0.45 | -0.28 | -0.28 | -0.07 | -0.20 | 0.06 | -0.35 | 0.06 | -0.67 | -0.17 | -0.86 | 1.00 | -0.24 | -0.83 | -0.15 | -0.83 |
| BIO16 | 0.01 | -0.38 | 0.10 | -0.47 | -0.35 | 0.33 | -0.77 | -0.21 | 0.28 | -0.21 | 0.28 | 0.88 | 0.98 | 0.56 | -0.24 | 1.00 | 0.64 | 0.98 | 0.64 |
| BIO17 | 0.10 | -0.64 | -0.33 | 0.01 | -0.05 | 0.34 | -0.40 | -0.07 | 0.11 | 0.07 | 0.11 | 0.90 | 0.59 | 0.99 | -0.83 | 0.64 | 1.00 | 0.60 | 1.00 |
| BIO18 | -0.01 | -0.28 | 0.22 | -0.60 | -0.43 | 0.31 | -0.85 | -0.28 | 0.32 | -0.29 | 0.32 | 0.83 | 0.98 | 0.51 | -0.15 | 0.98 | 0.60 | 1.00 | 0.60 |
| BIO19 | 0.10 | -0.64 | -0.33 | 0.01 | -0.05 | 0.34 | -0.40 | -0.07 | 0.11 | 0.07 | 0.11 | 0.90 | 0.59 | 0.99 | -0.83 | 0.64 | 1.00 | 0.60 | 1.00 |
